# Discovery of Sustainable Drugs for Neglected Tropical Diseases: Cashew Nut Shell Liquid (CNSL)‐Based Hybrids Target Mitochondrial Function and ATP Production in *Trypanosoma brucei*

**DOI:** 10.1002/cmdc.201800790

**Published:** 2019-02-05

**Authors:** Michela Cerone, Elisa Uliassi, Federica Prati, Godwin U. Ebiloma, Leandro Lemgruber, Christian Bergamini, David G. Watson, Thais de A. M. Ferreira, Gabriella Simões Heyn Roth Cardoso, Luiz A. Soares Romeiro, Harry P. de Koning, Maria Laura Bolognesi

**Affiliations:** ^1^ Department of Pharmacy and Biotechnology Alma Mater Studiorum – University of Bologna Via Belmeloro 6 40126 Bologna Italy; ^2^ Institute of Infection, Immunity and Inflammation University of Glasgow GBRC, University Place G12 8AT Glasgow UK; ^3^ Department of Biochemistry Faculty of Natural Sciences Kogi State University P.M.B. 1008 Anyigba Kogi State Nigeria; ^4^ Wellcome Trust Centre for Molecular Parasitology Institute of Infection, Immunity and Inflammation University of Glasgow GBRC, University Place G12 8AT Glasgow UK; ^5^ Strathclyde Institute of Pharmacy and Biomedical Sciences University of Strathclyde 16 Richmond Street G1 1XQ Glasgow UK; ^6^ Department of Pharmacy, Health Sciences Faculty University of Brasília Campus Universitário Darcy Ribeiro 70910-900 Brasília DF Brazil

**Keywords:** antiprotozoal agents, cashew nut shell liquid, hybrid drugs, natural products, trypanosomiasis

## Abstract

In the search for effective and sustainable drugs for human African trypanosomiasis (HAT), we developed hybrid compounds by merging the structural features of quinone **4** (2‐phenoxynaphthalene‐1,4‐dione) with those of phenolic constituents from cashew nut shell liquid (CNSL). CNSL is a waste product from cashew nut processing factories, with great potential as a source of drug precursors. The synthesized compounds were tested against *Trypanosoma brucei brucei*, including three multidrug‐resistant strains, *T. congolense*, and a human cell line. The most potent activity was found against *T. b. brucei*, the causative agent of HAT. Shorter‐chain derivatives **20** (2‐(3‐(8‐hydroxyoctyl)phenoxy)‐5‐methoxynaphthalene‐1,4‐dione) and **22** (5‐hydroxy‐2‐(3‐(8‐hydroxyoctyl)phenoxy)naphthalene‐1,4‐dione) were more active than **4**, displaying rapid micromolar trypanocidal activity, and no human cytotoxicity. Preliminary studies probing their mode of action on trypanosomes showed ATP depletion, followed by mitochondrial membrane depolarization and mitochondrion ultrastructural damage. This was accompanied by reactive oxygen species production. We envisage that such compounds, obtained from a renewable and inexpensive material, might be promising bio‐based sustainable hits for anti‐trypanosomatid drug discovery.

## Introduction

Neglected tropical diseases (NTD) are a group of 17 highly debilitating and potentially fatal poverty‐related diseases. These include protozoan, bacterial, and helminthic infections that prevail in tropical and subtropical areas in 149 countries. Communities living in poverty, lacking access to basic sanitation and in close contact with infectious disease vectors, domestic animals and livestock, are those worst affected.^1^ Notwithstanding the recent re‐emergence of interest in NTD, continuous research efforts are needed to sustain any drug development pipeline in the medium and long term.^2^

Human African trypanosomiasis (HAT) is one of the most neglected tropical diseases, endemic in sub‐Saharan Africa. *Trypanosoma brucei rhodesiense* (East and Southern Africa) and *T. b. gambiense* (West and Central Africa) are the causative protozoan parasites, which are transmitted to humans by tsetse flies that are found only in Africa.^3^ This disease, which is disabling and fatal if left untreated, is a major cause of rural underdevelopment and severely affects economies and communities. The control and elimination of HAT, which are declared goals of the WHO,^1^ would be a major step in the reduction of the overall burden of tropical disease that continues to limit development in sub‐Saharan Africa.^4^ Although HAT transmission is limited to the tsetse belt, comprising much of sub‐Saharan Africa, the risk of HAT in travelers and migrants, albeit low, cannot be overlooked.^5^ Moreover, animal African trypanosomiasis (AAT) has an enormous impact on African agriculture and food security. This condition is caused by related trypanosome species including *T. b. brucei*, *T. congolense*, *T. evansi*, and *T. vivax*, and, not necessarily being dependent on tsetse flies, has spread to much of South America, South Asia, and the Middle East.^6^

HAT treatment today relies on five drugs (pentamidine (PMD), suramin, melarsoprol, nifurtimox, and eflornithine; see Supporting Information Figure S1 for chemical structures), which suffer from toxic side effects, lack of efficacy, and development of resistance.^1^ The management of patients using these drugs is complex and risky, requiring the support services of a well‐trained staff.^7^ Moreover, despite the fact that almost all HAT control programs subsidize the cost of drugs and hospitalization,^8^ the availability of quality medicinal agents on a sustainable basis is an increasingly appreciated public health care concept. Consequently, lowering the costs of therapy by developing new drugs based on inexpensive resources is a valuable approach to be pursued.

Based on the above considerations, as well as on our continuous interest in the NTD field, we explored the possibility of using cashew nut shell liquid (CNSL) as a sustainable, low‐cost starting material for the development of new drugs against trypanosomiases. CNSL, which is obtained as the by‐product of cashew nut processing, has proven to be one of the most versatile food wastes for the production of functional materials and chemicals.^9^ However, its potential as a precursor of drugs has been relatively underexplored. Being an inedible waste material, it presents clear environmental, financial and ethical advantages over synthetic drugs and even over natural products derived from crops grown for that purpose.^10^ In addition, the fact that East Africa (Tanzania, Kenya, and Mozambique) and West Africa (Benin, Guinea‐Bissau, Ivory Coast, and Nigeria) are among the largest CNSL‐producing countries, opens up the exciting possibilities to engage endemic countries as crucial actors in NTD drug discovery and development.^11^ On this basis, we have developed a new chemical library of CNSL‐derived hybrids and investigated their anti‐trypanosomal potential. In particular, the compounds were evaluated against wild‐type (WT) and multidrug‐resistant African trypanosomes; *T. b. brucei* is extremely closely related to the human‐infectious species, and *T. congolense* is the principal agent causing AAT in Africa. Some of the compounds displayed low micromolar activity against *T. b. brucei* and absence of toxicity on a human cell line. We therefore investigated the mechanism by which this compound class exerts its trypanocidal activity.

## Results and Discussion

### Design rationale

**Figure 1:**
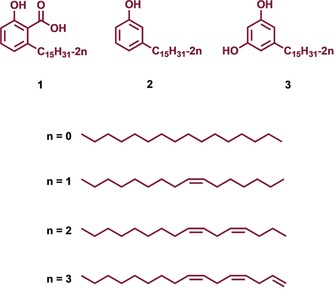
The main components of CNSL.

CNSL mainly consists of phenolic lipids, that is, anacardic acids (**1** in Figure 1) (71.7 %), cardanols (**2**) (4.7 %), and cardols (18.7 %) (**3**).^12^ The pentadecyl alkyl side chain of **1**–**3** may be saturated, mono‐olefinic, di‐olefinic or tri‐olefinic with a high percentage of the components having one or two double bonds (Figure 1), depending on the production method.^12^ Although CNSL components have been reported to possess a wide range of biological activities, in many cases they are not potent enough to be drug candidates.^9^ To overcome this limitation, their use in combination with standard drugs, and the design of new semi‐synthetic derivatives have been exploited.^9^

**Figure 2:**
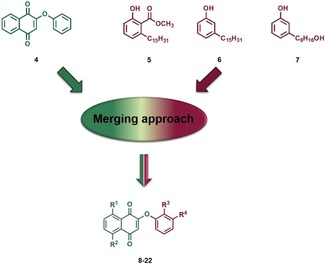
Design strategy to generate CNSL‐based hybrids **8**–**22** (see Scheme 1 for structures).

Along these lines, we decided to develop a series of CNSL‐based hybrid compounds. In particular, building on the strategy that the combinations of two different fragments into one covalently linked hybrid compound can convey synergy and increase potency,^13^ we combined the chemical features of CNSL derivatives with those of a previously developed anti‐trypanosomal hit compound (**4** in Figure 2).^14^ Intriguingly, both **4** 14b and a mixture of anacardic acids,^15^ isolated from Brazilian CNSL, have been reported to inhibit trypanosomal glyceraldehyde‐3‐phosphate dehydrogenase (GAPDH), an essential glycolytic enzyme and a validated anti‐trypanosomatid target.^16^

**Scheme 1:**
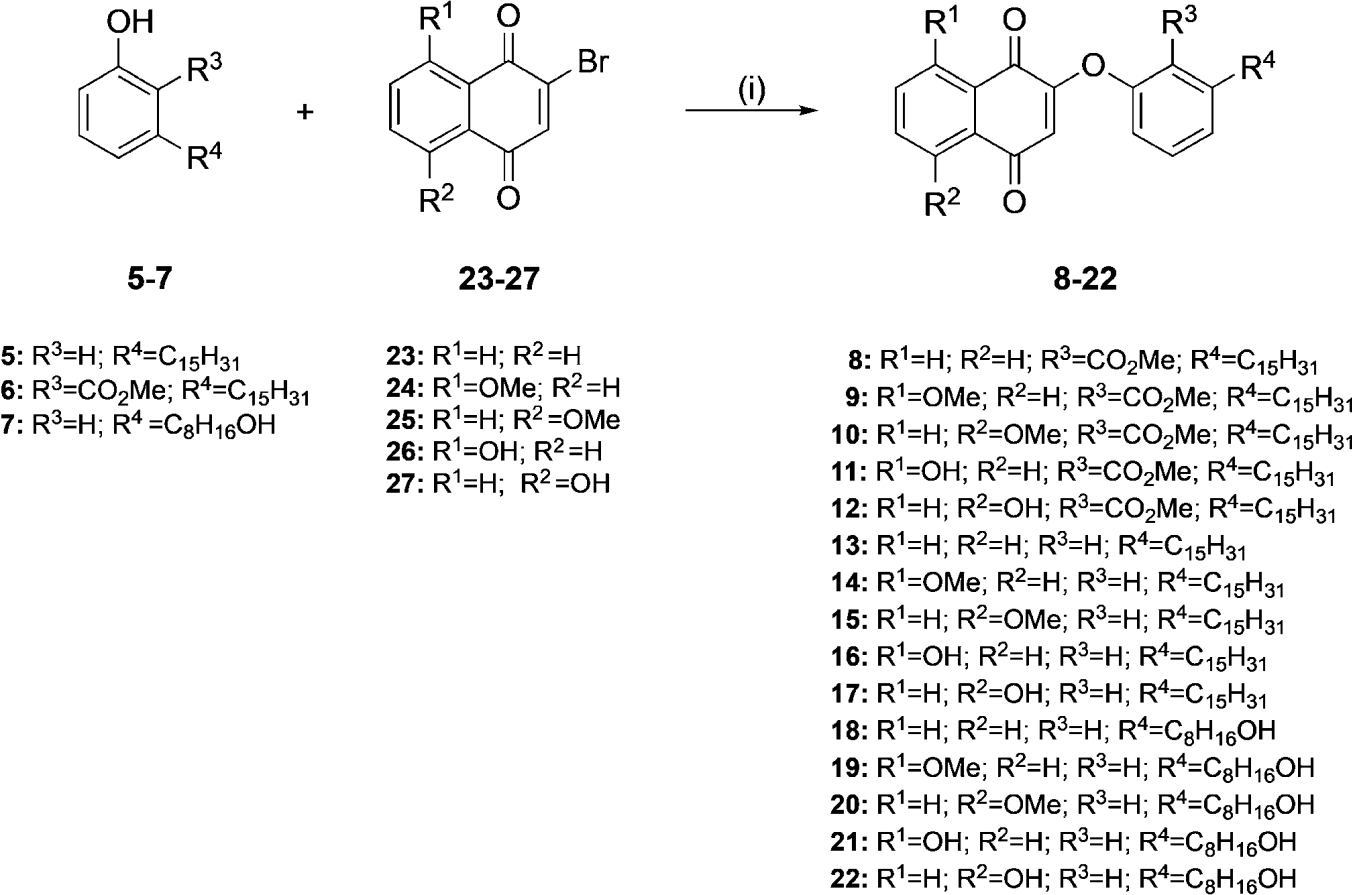
Synthesis of target compounds **8**–**22**: i) K_2_CO_3_, DMF, RT, 3 %–65 %.

Furthermore, thanks to the presence of a naphthoquinone moiety, **4** was shown to generate reactive oxygen species (ROS), a mechanism that may further contribute to its multi‐target trypanocidal activity.14b In fact, **4** exhibited high potency against *T. b. rhodesiense* (STIB 900 strain) (IC_50_=80 nm) and a promising selectivity index (SI) of 74, with respect to L6 mammalian cells.14a In particular, we anticipated that overlapping **4** with CNSL derivatives **5** and **6** (Figure 2) through their common phenoxy moiety could lead to hybrids with an improved anti‐trypanosomal profile and an improved sustainability. In addition, considering that the presence of the long alkyl chain (C_15_) might limit drug‐likeness due to excessive lipophilicity (see predicted physicochemical properties in Table 1 and S1) and might give rise to surfactant properties and nonspecific activities, we also turned our attention to the shorter‐chain (C_8_) CNSL derivative **7**. Following this design strategy, the small combinatorial library of **8**–**22** was generated (Figure 2 and Scheme 1).

**Table 1:** EC_50_ values [μm] against trypomastigotes of *T. b. brucei* (*T. b. b*.), *T. congolense* (*T. c*.), and HFF cells, along with selectivity indexes, resistance factors, and predicted log*P* values for compounds **8**–**22**.

Compd	*T. b. b*. 427WT	SI^[a]^	*T. b. b*. B48	RF^[b]^	*T. b. b*. aqp2/aq3‐KO	RF^[b]^	*T. c*. IL3000 WT	HFF^[c]^	log*P* ^[d]^
**8**	>200		n.d.		n.d.		n.d.	n.d.	10.88
**9**	>200		n.d.		n.d.		n.d.	n.d.	10.85
**10**	>200		n.d.		n.d.		n.d.	n.d.	10.85
**11**	>200		n.d.		n.d.		n.d.	n.d.	11.07
**12**	>200		n.d.		n.d.		n.d.	n.d.	11.07
**13**	>200		n.d.		n.d.		n.d.	n.d.	10.75
**14**	>200		n.d.		n.d.		n.d.	n.d.	11.0
**15**	>200		n.d.		n.d.		n.d.	n.d.	11.0
**16**	>200		n.d.		n.d.		n.d.	n.d.	11.22
**17**	>200		n.d.		n.d.		n.d.	n.d.	11.22
**18**	9.1±1.4	>21.98	8.6±2.4	0.95	10.1±2.2	1.1	n.d.	n.e.	5.64
**19**	15.0±1.3	>13.3	14.6±1.7	0.98	17.7±1.5	1.2	n.d.	n.e.	5.62
**20**	5.0±0.3	>40	5.3±0.8	1.1	6.2±0.58	1.2	18.8±0.2	n.e.	5.62
**21**	40.5±4.9	>5	36.9±5.3	0.91	46.7±7.7	1.2	n.d.	n.e.	5.84
**22**	7.6±0.7	>26.3	6.8±0.1	0.91	7.3±0.5	0.96	43.4±0.2	n.e.	5.84
**4**	48.7±0.8	n.d.	63.0±0.4	1.3	62.4±0.8	1.3	108±2	n.d.^[g]^	n.d.
PMD^[e]^	0.0093±0.0001		3.1±0.7	339	0.20±0.01	22.0	n.d.	n.d.	n.d.
DA^[f]^	n.d.		n.d.		n.d.		0.17±0.0003	n.d.	n.d.

None of the EC_50_ values for the test compounds against the *T. b. b*. 427WT strain were significantly different in the B48 or aqp2/aqp3‐KO strains. In contrast, the EC_50_ value for pentamidine (PMD) was highly significantly different in the latter two strains relative to 427WT (*p*<0.001, Student's unpaired t‐test, *n*=4 for all data points); n.d.: not determined; n.e.: no effect at 200 μm. [a] Selectivity index=EC_50_(HFF)/EC_50_(*T. b. b*. WT). [b] Resistance factor relative to WT. [c] Cytotoxic activity (EC_50_) on human foreskin fibroblast (HFF) cells; cytotoxic activity was observed up to 200 μm. [d] log*P* values were predicted with FAF*Drugs*4 software (http://fafdrugs3.mti.univ‐paris‐diderot.fr). [e] Pentamidine. [f] Diminazene aceturate. [g] The EC_50_ value of **4** is 9.84±3.25 against human dermal fibroblasts (HDF).14c

### Synthetic strategies for CNSL‐based hybrids (8–22) and CNSL‐phenols (5–7)

The synthesis of **8**–**22** was performed as illustrated in Scheme 1. We exploited a previously developed one‐pot reaction to combine phenols **5**–**7** and 2‐bromo‐1,4‐naphtoquinones **23**–**27**.14a,14c Phenols **5**–**7** were first treated with K_2_CO_3_ at room temperature in DMF, and then the proper 2‐bromo‐1,4‐naphtoquinone (**23**–**27**) was added, to rapidly (2–3 h) afford final compounds **8**–**22**. Despite its utility in nucleophilic substitution reaction, DMF is clearly not compatible with the current drive toward more sustainable and environmental friendly medicinal chemistry processes.^17^ With this in mind, we substituted DMF, which has been categorized as a hazardous solvent, with the safer DMSO.^18^ Encouragingly, similar yield were obtained (see Experimental Section). 2‐Bromo‐1,4‐naphthoquinone **23** was commercially available, whereas methoxy‐ and hydroxy‐naphthoquinones **24**–**27** were prepared as previously reported.^19^

**Scheme 2:**
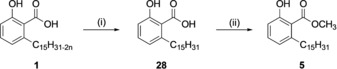
Synthesis of phenol **5**: i) H_2_, Pd/C, EtOH, RT, 70 %; ii) H_2_SO_4_, CH_3_OH, reflux, 86 %.

On the other hand, the CNSL‐based phenols **5**–**7** have been synthesized starting from the cashew nut shells. Particularly, to synthesize phenol **5**, an extraction of 150 g of cashew nut shells with ethanol yielded 60 g of natural CNSL (40 wt % based on the nut shells). The acids were then separated from other phenolic components by precipitation of their calcium salts using calcium hydroxide followed by acidification, which led to 21 g (70 wt % based on 30 g of the alcoholic extract) of the acid mixture **1**. Next, hydrogenation of **1** using palladium on carbon as catalyst allowed affording **28** in 70 % yield (Scheme 2).

**Scheme 3:**
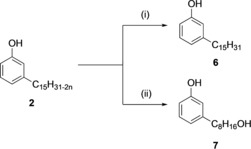
Synthesis of phenols **6**–**7**: i) H_2_, Pd/C, EtOH, RT, 90 %; ii) (a) Ac_2_O, H_3_PO_4_, MW, 400 W, 73 %, (b) O_3_, (CH_3_)_2_CO, −78 °C, (c) NaBH_4_, CH_3_OH, RT, 79 %.

Subsequently, **28** is converted into its corresponding methyl ester **5**, by performing a classical esterification reaction (H_2_SO_4_ in methanol). Toward the synthesis of CNSL‐based phenols **6** and **7**, our approach was based on distilled technical CNSL. Commercially available technical CNSL is obtained by treating hydrated shells at temperatures around 180–210 °C, and contains mainly the mixture of cardanols (**2**)—by decarboxylation of the anacardic acids (**1**)—and cardols (**3**). Distillation of the technical CNSL led to a heterogeneous mixture of cardanols **2**, as the primary component of CNSL. Hydrogenation of **2** using palladium on carbon as catalyst led to the saturated cardanol **6** in 90 % yield (Scheme 3). Conversely, compound **7** was synthesized using a three‐step protocol: a) protection of the phenol group by acetylation of **2**, b) oxidative cleavage by ozonolysis, c) reduction of the resulting secondary ozonide to the corresponding alcohol with sodium borohydride (Scheme 3).

### In vitro activity against *T. b. brucei* wild‐type and resistant strains and *T. congolense* wild‐type strain

The parent compound **4** and the CNSL‐based hybrid derivatives **8**–**22** were tested for effects on cell viability against bloodstream trypomastigotes of the standard drug‐sensitive *T. b. brucei* strain 427WT. The most active compounds were further tested against the multidrug‐resistant strain B48 and the drug‐transporter deletion mutant aqp2/aqp3‐KO (Table 1). The current first‐line drug PMD was used as the reference compound. It should be noted that the EC_50_ values reported herein were produced using a different species (*T. b. brucei* versus *T. b. rhodesiense*) and a different resazurin‐based protocol from that used in the previous report,14a in which the EC_50_ value of **4** against *T. b. rhodesiense* was found to be 80 nm. The very robust protocol used here uses a much higher cell density and consequently results in substantially higher EC_50_ values.^20^ Using this protocol, only compounds **18**–**22**, which carry the shorter (C_8_) aliphatic chain, showed anti‐trypanosomal activity in the micromolar range (5.0–40.5 μm). Importantly, under these conditions, **18**–**22** had higher activity than **4**, which displays an EC_50_ value of 48.7 μm. Crucially, compounds **18**–**22** showed no sign of human cytotoxicity: none of the compounds displayed any effects on either the viability or growth of the human foreskin fibroblast (HFF) cell line at the highest tested concentration (200 μm). Notably, **18**–**22** displayed statistically identical activity (*p*>0.05) against the aqp2/aqp3‐KO cell line from which the well‐characterized drug transporter HAPT1/TbAQP2 has been deleted,^21^ resulting in a moderated level of PMD resistance (Table 1). Indeed, there was no cross‐resistance detected even in the very highly multidrug‐resistant cell line B48, although it displayed 178‐fold resistance to PMD in this series of experiments (Table 1). Interestingly, compounds **18**–**22** were up to 12‐fold more active against all strains than the starting compound **4**, suggesting an improvement of the activity in at least some of the newly synthetized CNSL‐based hybrids. This shows that the shorter alkyl chain with a primary alcoholic end function, together with the regioisomeric substitution on the naphthoquinone moiety, enhances the anti‐trypanosomal activity of the test compounds (see below).

The two most active compounds **20** and **22** and the parent compound **4** were also tested against *T. congolense* IL3000 cell line and compared with the standard drug against animal trypanosomiasis, diminazene aceturate (DA in Supporting Information Figure S1). All three compounds were less active than DA, with EC_50_ values in the mid‐to‐high micromolar range, as well as severalfold less active than against *T. brucei*. Despite the small number of compounds tested, the results with *T. congolense* suggest that this species may be systematically less sensitive to this scaffold than *T. brucei* is. Similarly, *T. congolense* is less sensitive than *T. brucei* to suramin^6^ and PMD,^22^ reflecting species differences in drug target or accumulation.

### Structure–activity relationship (SAR) of trypanocidal activity

Our goal was to design and synthetize a small library of CNSL‐based hybrids, aiming for a synergistic inhibition of energy metabolism in *T. b. brucei*, targeting mitochondrial functions and GAPDH inhibition. Along the line of this rationale, we designed the library of CNSL‐based hybrids starting from quinone **4** and longer (**5** and **6**) and shorter (**7**) CNSL derivatives. Intriguingly, only C_8_ compounds **18**–**22** showed a promising anti‐trypanosomal activity, whereas **8**–**17** (C_15_) were not effective up to a concentration of 200 μm. Notably, a significantly higher efficacy was detected for **18**–**22** in comparison with **4**, suggesting that, at least for this subset, the proposed hybridization strategy was successful. As expected by the predicted log*P* values (Table 1), the nature of the chain dramatically affected activity, probably by modulating cell partitioning. This might be due to the insertion of the alkyl chain on the phenoxy ring that might positively modulate the lipophilicity of the test compounds increasing cell viability. In fact, significant differences can be appreciated among the longer‐ and the shorter‐chain subsets: the CNSL‐based derivatives **8**–**12** and **13**–**17** showed no anti‐trypanosomal activity against *T. b. brucei* up to 200 μm.

### Anti‐trypanosomal profile and time‐to‐kill determination

**Figure 3:**
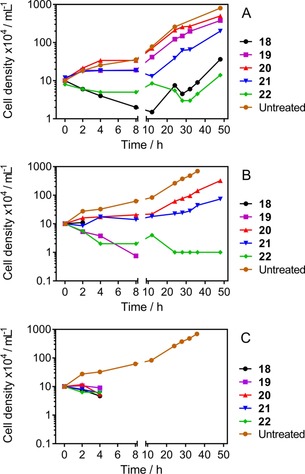
Cell counts of *T. b. brucei* cultures (bloodstream trypomastigotes), seeded at 1×10^5^ cells mL^−1^, incubated in the presence of either A) 0.5×, B) 1×, or C) 2× the EC_50_ of the test compounds. Where the lines stop, no live cells were detected at the next time point. Error bars represent SEM; where they are not shown, they are obscured by the symbols.

To determine whether **18**–**22** inhibited growth or cell division rather than causing cell death of *T. b. brucei*, cell growth curves were performed by treating 427WT trypomastigotes with concentrations corresponding to 0.5×, 1×, and 2× the EC_50_ value, using untreated cells as a control. Incubation with test compounds at half the EC_50_ (Figure 3 A) caused mostly some delayed growth phenotype, with rates increasing after 10 or 20 h. At 1× their respective EC_50_ (Figure 3 B), the hybrid compounds induced a consistent, rapid‐onset decrease in cell growth rate over 48 h. Compounds **18** and **19** showed trypanocidal activity at this concentration, sterilizing the culture in 2 and 8 h, respectively. Compound **22** appeared similarly to rapidly decrease the cell density but, at this concentration, killed only a proportion of the cell population before stabilizing; **20** and **21** decreased the growth rate substantially, with a nearly trypanostatic effect over the first 12 h (Figure 3 B). At double the concentration (Figure 3 C), all the hybrids cleared the culture between the 4 h and 8 h time points, with the cell populations rapidly declining between 2 and 4 h. The rapid time to kill displayed by **18**–**22** (and the lack of cross‐resistance with existing chemotherapy) is a clear advantage toward any (pre)‐clinical development of this scaffold, and encourages further hit optimization efforts.

### Activity of the compounds against *T. brucei* GAPDH

Based on the reported activity of starting compound **4**, which displays an IC_50_ of 7.25 μm against GAPDH,14b we first tested inhibition of this enzyme by **8**–**22**, at a fixed concentration of 10 μm, following a previously reported protocol.14c However, none of the compounds displayed significant inhibition of GAPDH activity (<15 % decrease observed, data not shown). One explanation for this lack of inhibitory activity might be that the current series of hybrids is partially or completely prevented from binding the active site of the trypanosomal glycolytic enzyme because of steric hindrance by the long alkyl chain. Limitations of solubility prevented us from testing the series at higher concentrations. However, it must be noted that we cannot exclude the possibility of a low‐affinity inhibition of GAPDH contributing to the trypanocidal effect, as compounds may accumulate to relatively high concentrations within the parasite. Indeed, examples where a high level of accumulation include almost all the first‐line trypanocidal agents (Supporting Information Figure S1), including DA,^23^ PMD,^24^ melarsoprol,^25^ and suramin^26^ and highly active experimental therapies, including a recently described series of bisphosphonium compounds^27^ that strongly accumulate in the *T. b. brucei* mitochondrion.

### Mode of action studies

Because the drug design of our series of hybrids is based on the naphthoquinone framework that is recognized as a privileged structure for the modulation of the mitochondrial functions^28^ and **4** itself acted at mitochondrial targets through production of ROS,14b we had reason to further investigate their mode of action at the mitochondrial level in *T. b. brucei*. Furthermore, the chemical structures of the newly CNSL‐based hybrids resemble that of ubiquinone (Supporting Information Figure S2), which is an essential carrier in the electron‐transport chain via a redox reaction inner the mitochondrial membrane. Accordingly, we initially aimed for a synergistic inhibition of the energy metabolism for our hybrids, targeting the mitochondrial membrane enzymes involved in electron transport (e.g., trypanosome alternative oxidase (TAO), F_o_F_1_‐H^+^ ATPase), in addition to glycolysis (GAPDH, located in the glycosome).^29^ This because inhibition of both of the essential arms of trypanosomal energy metabolism would be expected to deliver synergistic effects. Despite the fact that we were not able to demonstrate a GAPDH inhibitory activity of our hybrids, we looked at the effects of the two most active compounds **20** and **22** on ATP content and on the mitochondrial membrane potential (MMP), as relevant parameters for an action on the trypanosomal energy metabolism.^30^

### ATP and MMP determination as relevant evidence for a mitochondrial mode of action

**Figure 4:**
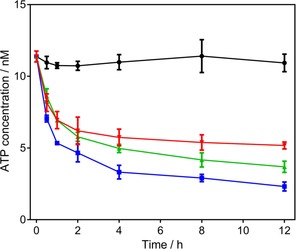
ATP content of bloodstream trypomastigotes of *T. b. brucei* 427WT after incubation with oligomycin (2.0 μg mL^−1^ ▪), **20** (2.2 μm ▾), **22** (3.5 μm ▴) or no drug (control •). Data are the average±SEM of three determinations.

**Figure 5:**
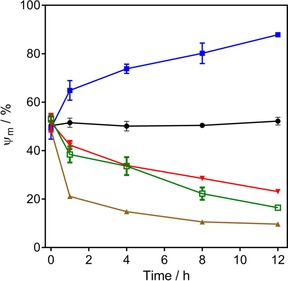
Mitochondrial membrane potential *Ψ*_m_ in bloodstream trypomastigotes of *T. b. brucei* 427WT was determined at various time points after incubation with various test compounds: troglitazone (10 μm ▪), valinomycin (0.1 μm ▴), **20** (2.4 μm ▾), **22** (3.5 μm □), or untreated (control •). *Ψ*_m_ values are expressed as the percentage of cells with 500 or more artificial units of fluorescence, calibrated at 50 % for the control group at *t*=0 h. Data are the average±SEM of three independent experiments. Valinomycin and troglitazone were used as controls for depolarization and hyperpolarization, respectively.

*T. b. brucei* 427WT BSF were incubated with **20** and **22**, at approximately 0.5×EC_50_, and the ATP content was determined at different time points (Figure 4). The F_o_F_1_‐ATPase inhibitor oligomycin (see Supporting Information Figure S2 for structure) was used as positive control and untreated cells as negative control. We found that both compounds rapidly decrease the ATP content, with even 30 min inducing a highly significant reduction in cellular ATP levels (*p*<0.01), stabilizing at approximately 50 % of untreated [ATP] after 4–6 h (*p*<0.001). The response to **20** and **22** was similar not just to each other, consistent with an identical mode of action, but also highly similar to that of oligomycin, although the latter depressed the ATP levels even further at the respective concentrations used (Figure 4). It should be noted that the observed decrease in ATP does not correlate with cell death, which even at 2×EC_50_ does not occur in large number until after 4 h. The observed decrease in ATP levels could be the result of mitochondrial functions and therefore we next investigated the timing of the same concentrations of **20** and **22** on the mitochondrial membrane potential *Ψ*_m_, using flow cytometry with the fluorescent probe TMRE. Valinomycin (Supporting Information Figure S2) was used as the control for depolarization and troglitazone (Supporting Information Figure S2), as the control for hyperpolarization (Figure 5).^31^

Both test compounds decreased *Ψ*_m_ slightly over the first hour (*p*>0.05), an effect that was stronger at the 4 h (*p*<0.05 for **20**) and subsequent time points (*p*<0.01 for both compounds). The *Ψ*_m_ is expressed as the percentage of cells displaying a fluorescence of ≥500 arbitrary units (calibrated at 50 % for untreated cells at *t*=0), with a shift toward lower fluorescence indicating a mitochondrial membrane depolarization (Supporting Information Figure S3). It is clear from the time dependency and magnitude of the effects that cellular ATP depletion preceded the partial depolarization of the mitochondrial inner membrane. We thus conclude that the depolarization is likely to be the result of the decreased availability of ATP for the F_o_F_1_‐ATPase, which maintains the membrane potential of the mitochondrion of bloodstream *T. brucei*, using the ATP to pump protons out of the mitochondrial matrix.^32^ The primary cause of the ATP depletion may be the inhibition of an essential mitochondrial function, and/or a step in the glycolysis.

However, we re‐tested compounds **18**, **19**, **20** and **22** against 427WT cells, in the presence and absence of 5 mm glycerol in the medium, an addition that sensitizes the cells to TAO inhibitors such as salicylhydroxamic acid (SHAM) and ascofuranone (Supporting Information Figure S2).^33^ Indeed, we found that the cells were significantly sensitized to SHAM in the presence of glycerol (*p*<0.001), but not to the hybrid compounds (Table 2). Interestingly, compound **20** displayed significantly (*p*<0.001) less activity in the presence of glycerol, whereas the EC_50_ values for the other test compounds was unchanged. We conclude that **18**, **19**, **20** and **22** do not act via direct inhibition of TAO.

**Table 2:** Investigation of the role of TAO in the trypanocidal activity of test compounds.

Compd	Control s427WT	Control s427WT + 5 mm glycerol		
	EC_50_ [μm]^[a]^	EC_50_ [μm]^[a]^	RF^[b]^	*p* value^[c]^
**18**	30.0±0.08	31.2±0.6	1.04	n.s.
**19**	16.8±0.06	16.3±0.4	0.97	n.s.
**20**	9.2±0.04	16.0±0.2	1.74	*p*<0.001
**22**	25.4±1.0	28.2±0.2	1.11	n.s.
SHAM^[d]^	86.3±0.4	58.9±0.6	0.68	*p*<0.001

[a] Values are the average±SEM of three independent determinations. [b] Resistance factor. [c] Statistical significance was determined using Student's unpaired t‐test; n.s.: not significant. [d] Salicylhydroxamic acid.

### CNSL‐based hybrids increase the production of reactive oxygen species (ROS) in *T. b. brucei*

**Figure 6:**
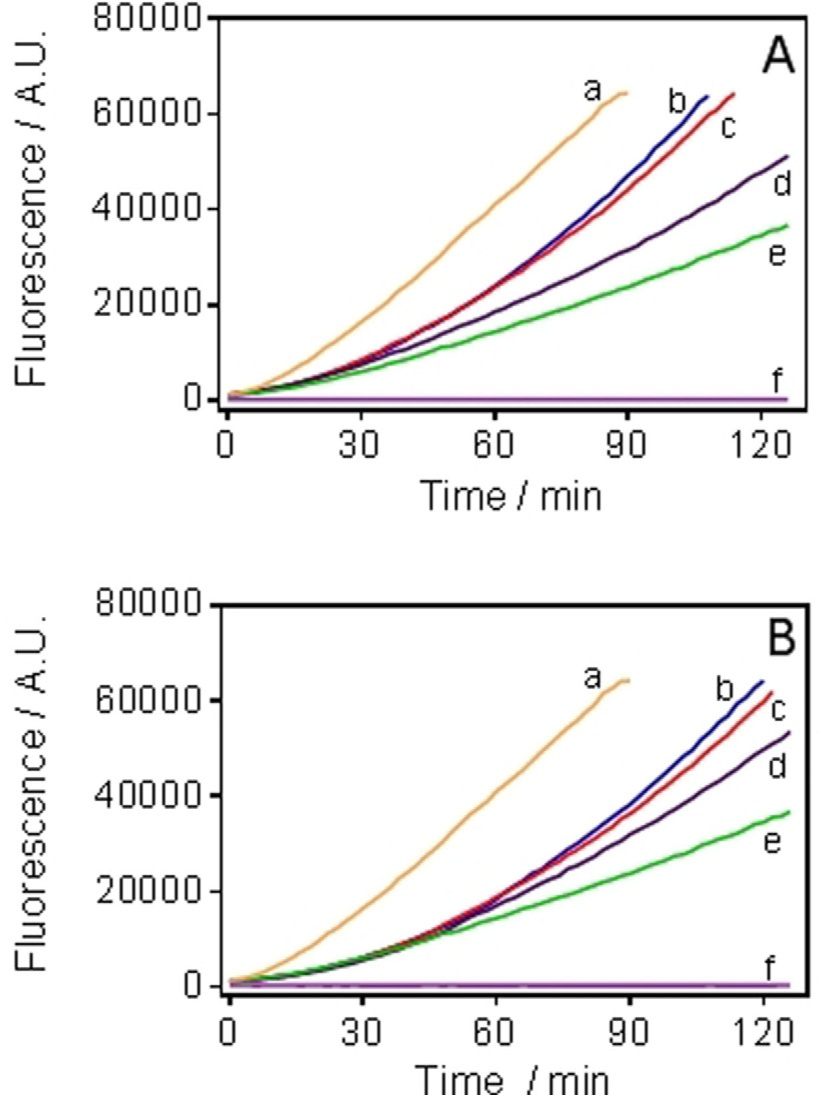
Effect of A) **20** and B) **22** on ROS production in bloodstream forms of *T. b. brucei* s427WT. Production of ROS was measured using the fluorescent indicator dye DCFH‐DH. Cells incubated with 100 μm H_2_O_2_ (a); CNSL‐based hybrids were at: 2×EC_50_ (b), 1.25×EC_50_ (c), and 0.32×EC_50_ (d). Parasites incubated without test compound (e); plain assay buffer without cells (f).

To determine whether our hybrids display a redox activity in *T. b. brucei* 427WT BSF (similarly to what has been demonstrated for **4**),14b we assessed the production of ROS in cells treated with **20** and **22** and in control cells, using the ROS‐sensitive fluorescent dye 2′,7′‐dichlorodihydrofluorescein diacetate (DCFH‐DH) (Figure 6).^34^

The results show that under normal culture conditions trypanosomes generate a steady amount of ROS, which is greatly increased in the presence of H_2_O_2_. The level of ROS was dose‐dependently increased over an incubation period of up to 2 h with **20** and **22** (Figure 6 A,B, respectively). As suggested previously for **4**,14b **20** and **22** could be substrates of the electron‐transport chain, leading to cycle of reduction by glycerol‐3‐phosphate dehydrogenase (G3PD) followed by reaction with molecular oxygen and the production of ROS. In normal conditions G3PD works like a shuttle between glycosomes and mitochondria, keeping the redox balance and feeding the respiratory chain for the ATP production,^35^ with TAO acting as electron acceptor, oxidizing the ubiquinol pool formed in consequence of G3PD activity.^36^ Accordingly, the observed overproduction of ROS is consistent with an ubiquinol‐like reactivity for our hybrids.^37^ This would cause both molecular and structural damage to *T. b. brucei* mitochondria (see section on Transmission Electron Microscopy, below).^38^

### CNSL‐based hybrids cause damages of the mitochondria but not the kinetoplast

**Figure 7:**
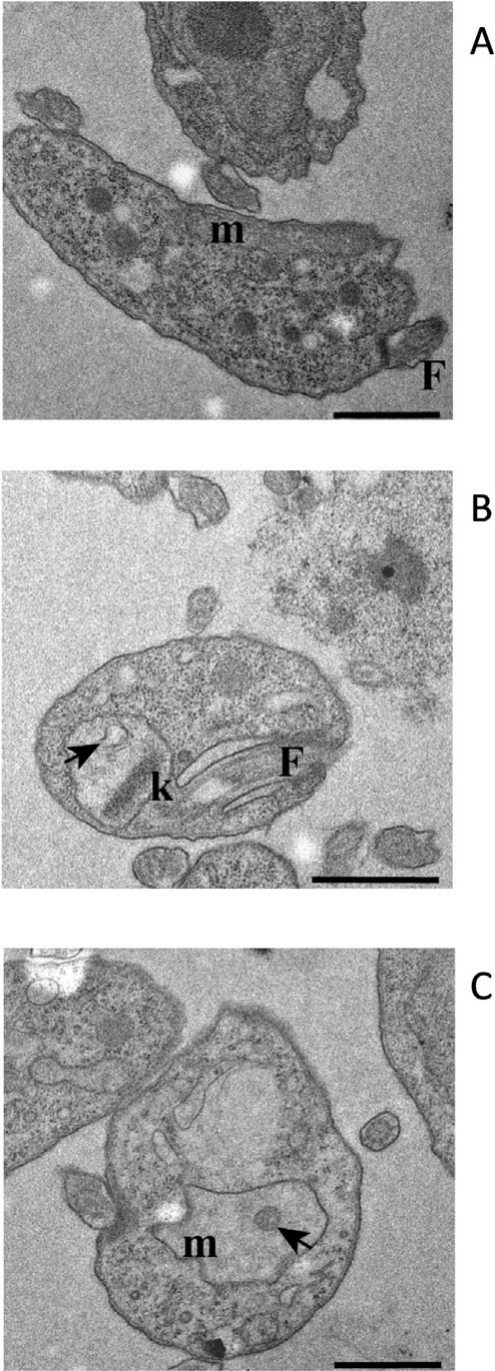
Ultrastructure TEM images of untreated (A) and treated (B, 4 h; C, 8 h) s427WT BSF with **22** at 1×EC_50_. m: mitochondrion, N: nucleus, F: flagellum, k: kinetoplast, arrow: membranous structure within the mitochondrion matrix. Scale bars: 1 μm.

Transmission electron microscopy (TEM) was used to study the trypanosomes’ ultrastructure after exposure to compound **22** at 1×EC_50_ (7.6 μm) in order to visualize any damage to the mitochondrion. Based on the information obtained from the ATP and *Ψ*_m_ determinations, TEM samples were taken after 4 and 8 h exposure to test compound, as “early” and “late” time points to define the effects on cellular ultrastructure (Figure 7). After 4 h of incubation with **20**, some mitochondria presented an irregular shape. Moreover, membranous, electron‐light structures had appeared in many mitochondria, resembling vacuoles (Figure 7, second row of images); no other ultrastructural changes were evident, appearing to confirm the mitochondrion as a main target for CNSL‐based hybrids in trypanosomes. Indeed, **20** caused further ultrastructure abnormalities in the mitochondrial structure at 8 h, displaying damage to the mitochondrial membrane, and the presence of membranous and dense vesicles within the matrix (Figure 7, third row). However, the treated cells do not display any morphological alteration to kinetoplasts, which completely maintain the original disk‐like structure.^39^ Accordingly, this evidence stands for an exclusive metabolic effect at mitochondrial level for the test compounds, which do not interfere with both functioning and replication of kinetoplasts.

**Figure 8:**
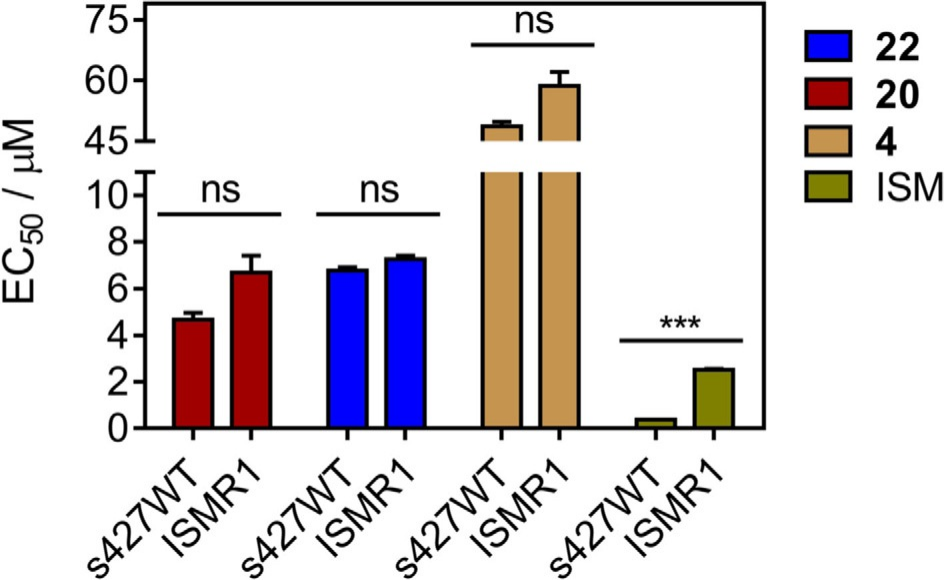
Comparison of the EC_50_ values for **20**, **22**, **4**, and the control drug isometamidium (ISM) obtained from the in vitro activity assay against bloodstream trypomastigotes of *T. b. brucei* s427WT and *T. b. brucei* ISMR1. Data are the average±SEM of three independent experiments; ns: not significant, ***p<0.001 (Student′s t‐test, unpaired).

To confirm whether the CNSL‐based hybrids exclusively target the mitochondrial energetic pathway rather than the mitochondrial kDNA, we decided to test compounds **4**, **20** and **22** against the isometamidium‐adapted ISMR1 cell line of *T. b. brucei* in parallel with the parental cell line 427WT. As ISMR1 is a dyskinetoplastic cell line, and thus highly resistant to the drug isometamidium (ISM; Supporting Information Figure S2) and other kinetoplast‐targeting drugs,^32^ a primary effect on energy metabolism rather than mitochondrial kDNA should not result in resistance to the test compounds. Indeed, compounds **4**, **20** and **22** did not show a significant increase in the EC_50_ values relative to the standard wild‐type strain 427, tested in parallel (Figure 8). These data exclude kDNA as a potential target for our test hybrids, with the available data all consistent with a mitochondrial target.

**Figure 9:**
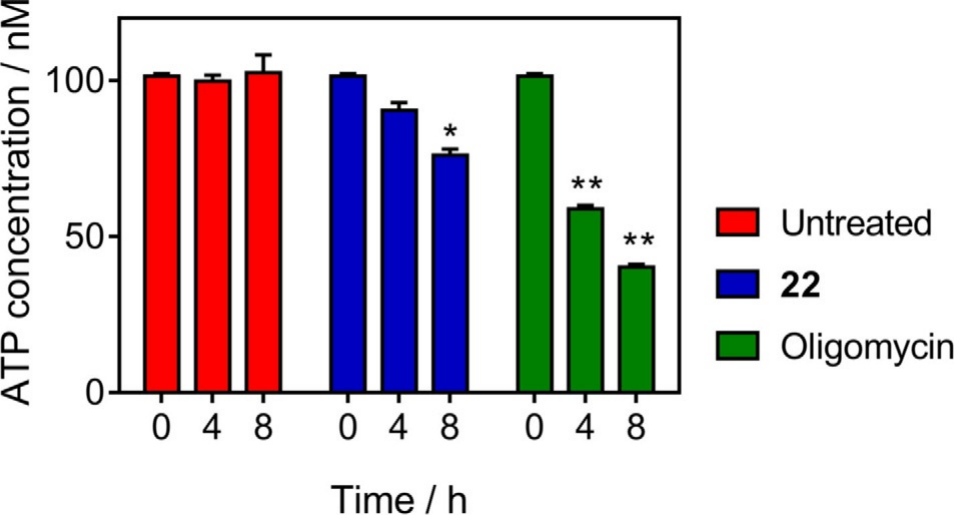
Determination of ATP content at various time points (0, 4, and 8 h) in untreated and treated HFF cells with compound **22** (200 μm). Detection was carried out using the firefly luciferase kit and measuring the levels of luminescence to calculate the concentration of ATP (nm). Oligomycin (2 μg mL^−1^) was used as positive control for decreasing ATP. Data are the average±SEM of three independent experiments; *p<0.05, **p<0.01 (Student′s t‐test, unpaired).

Although we had observed no toxicity of these naphthoquinone hybrids toward human cells, we next assessed whether they might damage mitochondria in human cells. We therefore determined the ATP content in treated and untreated HFF cell line with a deliberately high concentration (200 μm) of **22**. The compound had only a very minor effect on cellular ATP levels, even at this concentration, being approximately 26× trypanocidal EC_50_, although the effect became significant at 8 h of incubation (*p*<0.05). Oligomycin exhibited a strong effect even at 4 h (*p*<0.0.1) (Figure 9).

**Figure 10:**
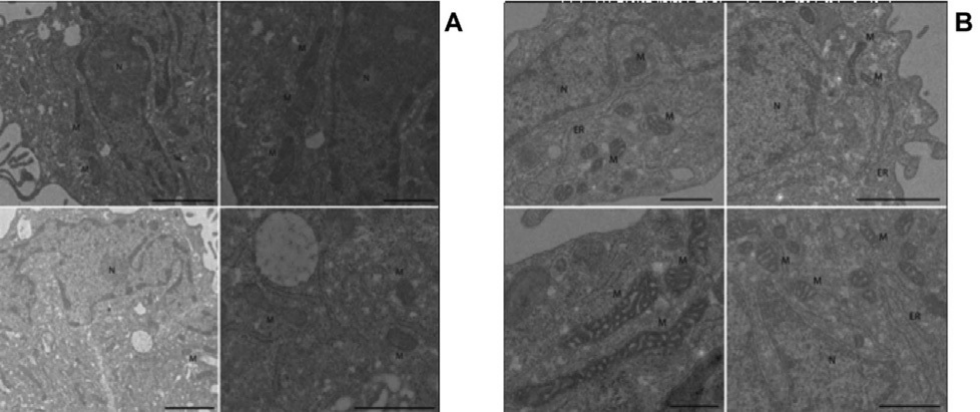
TEM images of HFF cells cultured for 12 h in the absence (A) or presence (B) of compound **20** at 200 μm. ER: endoplasmic reticulum, N: nucleus, M: mitochondrion.

We also used TEM after exposure to the same concentration of **22** (200 μm) at two different time points (4 and 12 h) in order to analyze whether ultrastructural changes similar to those in *T. brucei* could be observed. The TEM images reveal that no morphological damage occurs to mitochondria in human cells after treatment with even high concentrations of **22**, nor were any other ultrastructural changes in the exposed HFF cells visible (Figure 10). These results clearly show the absence of metabolic and structural toxic effects at the human mitochondrial level, indicating a highly species‐specific mode of action for our CNSL‐based hybrids.

### Metabolomics analysis

Metabolomics has successfully been used to elucidate the mode of action of drugs against *T. brucei*.^40^ We therefore used a metabolomics approach to further investigate the mode of action of **22**. *T. b. brucei* 427WT BSF were incubated with and without 0.5×EC_50_ of **22** for 2 h to identify specific metabolic changes caused by moderate exposure to the compound (early effects). Samples were instantly cooled down to block the intracellular metabolic activity, and processed to extract the pool of intracellular metabolites. Analysis by LC–MS showed a large number of metabolite differences between the treated and untreated cells, but without a clear pattern designating a particular enzyme function or pathway, or indeed a particular class of metabolites being affected apart from some sphingolipid precursors which accumulated in the exposed cells (Table 3), possibly indicating some direct or indirect interference in this pathway. These results are consistent with a multi‐target mode of action (as reported for **4**)14b or the result of general damage such as caused by ROS, affecting many cellular functions at once.

**Table 3:** Sphingolipid precursors elevated in trypanosomes treated with **22** versus untreated controls.^[a]^

*m*/*z*	*t*_R_ [min]^[a]^	Metabolite	*p* value^[b]^	Treated/Control
302.3046	5.0	sphinganine	0.034	1.654
538.5181	3.9	[SP (16:0)] *N*‐(hexadecanoyl)sphing‐4‐enine	0.013	2.450
540.5348	5.1	[SP (16:0)] *N*‐(hexadecanoyl)sphinganine	0.051	14.230
566.5504	3.8	[SP (18:0)] *N*‐(octadecanoyl)sphing‐4‐enine	0.009	3.028
566.5493	5.6	[SP (18:0)] *N*‐(octadecanoyl)sphing‐4‐enine	0.046	4.689
578.5528	3.9	sphing‐8(*Z*)‐enine‐19:0, ceramide	0.018	6.708

[a] Retention time. [b] Statistical significance was determined using Student's unpaired two‐tailed t‐test.

## Conclusions

In this report, we explore the use of a waste product of food production, the CNSL, as a cheap and abundant source of new anti‐infective agents for use against NTD. The required chemistry is simple, accessible and easily scaled up, making the entire production process highly affordable and local. Taken together, the collected data suggest that, in the case of compounds **20** and **22**, this CNSL hybridization strategy led to an increased trypanocidal activity up to 10‐fold relative to the parent compound **4**. This did not a priori signify that the new compounds necessarily act similarly as **4**, as the fusion with the CNSL‐based fragments affects both the pharmacokinetic and pharmacodynamic features of the synthesized hybrids. In addition to the contribution in target recognition, the long alkyl chain clearly affects the physicochemical properties, which are determinant for uptake by both target and host cells, delivery to mitochondria, and interaction with molecular targets.

We present clear evidence for mitochondrial targeting in trypanosomes, especially the TEM pictures are convincing, with mitochondrial damage but no other ultrastructural changes observed after just 4 h incubation with 1×EC_50_ of **22**. It is quite possible that this localized damage is linked to the observed production of ROS. However, ATP depletion precedes mitochondrial abnormalities and the depolarization of *Ψ*_m_ can be observed within 30 min, and we propose that the hybrids, as intended, exert more than one trypanocidal effect, consistent with the complex return of the metabolomics experiment, (although it was not possible to derive any clear conclusion from the metabolomics data, at this point). All this does not exclude a target inside the mitochondrion, but it is not (principally) TAO or glycerol‐3‐phosphate dehydrogenase (G3PDH), which together enable the re‐oxidation of glycolysis‐produced NAD^+^ via the cyanide‐resistant, non‐protonmotive oxidation of ubiquinol by TAO.^41^ This is because the activity of the hybrids was not enhanced in the presence of glycerol. The target linked to rapid ATP depletion could also be in glycosomes but we saw no evidence of glycosomal damage in the TEM images, nor consistent inhibition of GAPDH. The combination of cellular damage by ROS, while depleting the ATP required for damage repair, is likely to contribute to the rapid cell death observed at just 1–2×EC_50_.

It is probably because the kinetoplast is not involved in the trypanocidal action of these hybrid compounds that there is no cross‐resistance with diamidines (PMD and DA) and phenanthridine (ISM, ethidium) drugs. As these are the mainstay of human (PMD) and especially veterinary trypanosomiasis treatment, this is an important “plus” for this compound series. Even more important, the compounds displayed excellent in vitro selectivity over human cells; in addition to the lack of effect on growth rate of human fibroblasts, no effects on mitochondrial ultrastructure or ATP content, even at very high concentrations of compound were observed. This is especially encouraging in light of the severe toxicity of current HAT chemotherapy (Supporting Information Figure S1).^42^

In conclusion, while further rounds of optimization are required before these molecules can be turned into valuable leads for trypanosomiasis, we have successfully demonstrated that CNSL hybrids show promise as a template for the development of anti‐infective drugs on a sustainable basis. Importantly, the use of CNSL and its components for combating trypanosomiasis could be of high importance and economically feasible in developing countries that cultivate and process cashew and, at the same time, represent endemic areas. Further studies will focus on extending SAR, optimizing for *T. congolense*, and testing on veterinary trypanosome species that are more closely related to *T. brucei*: *T. evansi* and *T. equiperdum*, which, being dyskinetoplastic, are not treatable with DA or ISM, and not geographically restricted to the tsetse belt of Africa.^6^

## Experimental Section

### Chemistry

**General**: All of the commercially available reagents and solvents were used as purchased from Sigma–Aldrich and Tedia without further purification. The cardanol mixture (**2**) was purchased from Resibras (Fortaleza, Brazil). Reactions were followed by analytical thin‐layer chromatography (TLC), performed on precoated TLC plates (layer 0.20 mm silica gel 60 with a fluorescent indicator UV254, from Merck and F_254_ Silicycle plates). Developed plates were air‐dried and analyzed under a UV lamp (UV 254/365 nm) or visualized by exposure to iodine stain. The oxidative cleavages were performed using a Brasil Ozônio BrO3 ozonizer, whereas the catalytic hydrogenations were performed using a Parr Shaker Hydrogenation Apparatus 3916. Column chromatography purifications were performed using Sigma–Aldrich silica gel grade 9385, 60 Å, 230–400 mesh and Silicycle silica gel type G60, 60 Å, 70–230 mesh. Melting points were determined on a Quimis MQAPF 302 apparatus and are uncorrected. IR spectra were obtained on a PerkinElmer Spectrum BX infrared spectrophotometer. NMR experiments were run on Varian VXR 400 (400 MHz for ^1^H; 100 MHz for ^13^C) and on Bruker Avance DRX500 and DRX300 (500 and 300 MHz for ^1^H; 125 and 75 MHz for ^13^C, respectively) instruments. ^1^H and ^13^C NMR spectra were acquired at 300 K using deuterated chloroform (CDCl_3_) as solvent. Chemical shifts (*δ*) are reported in parts per million (ppm) relative to the residual solvent peak as internal reference, and coupling constants (*J*) are reported in hertz (Hz). The spin multiplicities are reported as s=singlet, br s=broad singlet, d=doublet, dd=doublet of doublet t=triplet, q=quartet, and m=multiplet. Mass spectra were recorded on a Waters ZQ 4000 apparatus, and electrospray ionization (ESI) in positive mode was applied. Compounds were named following IUPAC rules as applied by ChemDraw Professional (version 15.0). All tested compounds possess a purity of at least 95 % as assessed by HPLC. HPLC parameters were the following: MeOH (eluent A) and H_2_O with 0.05 % trifluoroacetic acid (eluent B); flow rate 1.0 mL min^−1^; elution type isocratic with 50 % of eluent A and 50 % of eluent B; detection UV/Vis Abs at 254 nm; Kinetex 5 μm EVO C_18_ 100 Å, LC Column 150×4.6 mm (see section Compound Purity and Table S2 in the Supporting Information for details).

#### Synthesis of phenols 5–7

**Natural CNSL extraction**: In a Soxhlet extraction system were added 150 g of cashew nutshell and absolute ethanol (400 mL). The system was maintained at reflux for 4 h. Then, the collecting flask was changed and a new extraction was carried out under the same conditions. In both cases, the solvent was evaporated under reduced pressure, providing 60 g of natural CNSL as a brown liquid in 40 % yield relative to the nutshell mass.

**Mixture of anacardic acids (1) from natural CNSL**: In a flask were added 30 g of natural CNSL (≈86.08 mmol), 15 g Ca(OH)_2_ (202.44 mmol) and methanol (180 mL) and water (30 mL). The reaction system remained at reflux and agitation for 3 h. Then, the mixture was cooled to room temperature and filtered. The solid obtained was washed with ethyl acetate to remove the other components of the CNSL. The calcium salts formed were treated with 50 % hydrochloric acid solution to pH 1.0 to liberate the mixture of anacardic acids, which were extracted with ethyl acetate (3×50 mL). The combined organic phases were washed with brine and dried over anhydrous sodium sulfate. After removal of the solvent under reduced pressure, the mixture was purified on a silica gel chromatography column, eluted in a mixture of hexane and 20 % ethyl acetate, affording the mixture of anacardic acids (**1**) as a brown oil in 70 % yield.

**2‐Hydroxy‐6‐pentadecylbenzoate (29)**: To a solution of heterogeneous mixture of anacardic acids **1** (5 g, 14.35 mmol for average molecular wild‐type 344) in ethanol (50 mL), was added with 10 % palladium on carbon (0.20 g, 2 mol %) and shaken in a Parr apparatus, under hydrogen atmosphere (60 psi) during 6 h. After, the reaction mixture was filtered in a sintered funnel and the filtrate was concentrated under reduced pressure. The residue was purified by chromatography with silica gel (hexane and then hexane/ethyl acetate 20 %) as eluent, to afford the title compound **6** as a white solid. Yield: 70 %; mp: 81–83 °C. IR (KBr): $\tilde{\nu }$_max_=3326 (*ν*_OH_); 2954 (*ν*_asCH3_); 2920 (*ν*_asCH2_); 2850 (*ν*_sCH2_), 1610 (*ν*_C=O_), 1560, 1542, 1498 and 1466 (*ν*_C=C_); 1287 (*ν*_asC−O_); 1086 cm^−1^ (*ν*_sC−O_). ^1^H NMR (300 MHz, CDCl_3_): *δ*=0.89 (t, *J*=6.5 Hz, 3 H); 1.26 (m, 24 H); 1.57–1.62 (m, 2 H); 3.00 (t, *J*=7.7 Hz, 2 H); 6.79 (d, *J*=7.4 Hz, 1 H); 6.89 (d, *J*=8.01 Hz, 1 H); 7.37 ppm (t, *J*=7.9 Hz, 1 H). ^13^C NMR (75 MHz, CDCl_3_): *δ*=14.3, 22.9, 29.6–30.0, 32.1, 32.2, 36.7, 110.6, 116.1, 123.0, 135.6, 148.1, 163.8, 176.5 ppm.

**Methyl 2‐hydroxy‐6‐pentadecylbenzoate (5)**: A mixture of 0.2 g of **28** (0.574 mmol) and 1.5 mL of H_2_SO_4_ in methanol was stirred and held at reflux for 18 h. After, the reaction was washed with brine (10 mL), extracted with dichloromethane (3×10 mL) and dried with anhydrous sodium sulfate. The solvent was evaporated under reduced pressure and the product was purified by chromatography on silica gel (hexane and then hexane/ethyl acetate 20 %) to afford the title compound **5** as a white solid. Yield: 86 %. IR (KBr): $\tilde{\nu }$_max_=3433 (*ν*_OH_), 2917 (*ν*_as CH2_), 2851 (*ν*_S CH2_), 1663 (*ν*_C=O_), 1451 (*ν*_C=C_), 1250, 1203 cm^−1^ (*ν*_as C−O_). ^1^H NMR (500 MHz, CDCl_3_): *δ*=0.89 (t, *J*=6.8 Hz, 3 H), 1.27–1.32 (m, 29 H), 1.51–1.55 (m, 2 H), 2.89 (t, *J*=7.8 Hz, 2 H), 3.96 (s, 1 H), 6.73 (d, *J*=7.3 Hz, 1 H), 6.84 (d, *J*=8.1 Hz, 1 H), 7.27–7.31 (m, 1 H), 11.06 ppm (s, 1 H). ^13^C NMR (125 MHz, CDCl_3_): *δ*=14.2, 22.9, 29.5–30.1, 32.1, 32.3, 36.8, 52.3, 112.0, 115.8, 122.6, 134.3, 146.4, 162.8, 172.1 ppm.

**3‐Pentadecylphenol (6)**: To a solution of heterogeneous mixture of cardanols **2** (monoene, diene, and triene (10 g, 33.05 mmol for average molecular wild‐type 300) in ethanol (40 mL), was added with 10 % palladium on carbon (0.25 g, 2 mol %) and shaken in a Parr apparatus, under hydrogen atmosphere (60 psi) during 4 h. After, the reaction mixture was filtered in a sintered funnel and the filtrate was concentrated under reduced pressure. The residue was purified by chromatography with silica gel using a mixture of hexane/dichloromethane (1:1) as eluent, to afford the title compound **6** as a white solid. Yield: 90 %; mp: 44–45 °C. ^1^H NMR (500 MHz, CDCl_3_): *δ*=0.92 (t, *J*=6.8 Hz, 3 H), 1.29–1.33 (m, 24 H), 1.60–1.61 (m, 2 H), 2.58 (t, *J*=7.7 Hz, 2 H), 6.67–6.69 (m, 2 H), 6.79 (d, *J*=7.4 Hz, 1 H), 7.16 ppm (t, *J*=7.7 Hz, 1 H). ^13^C NMR (125 MHz, CDCl_3_): *δ*=14.3, 22.9, 29.6–29.9, 31.5, 32.2, 36.0, 112.8, 115.6, 121.2, 129.5, 145.2, 155.6 ppm.

**3‐(8‐Hydroxyoctyl)phenol (7)**: An Erlenmeyer flask containing a solution of 12 g of mixture of cardanols **2** (monoene, diene and triene) (39.4 mmol) distilled acetic anhydride (12 mL) and phosphoric acid (12 drops) was placed inside an unmodified household microwave oven and irradiated for 3 min (3×1 min) at a power of 400 W. After, the residue was extracted with ethyl acetate (3×15 mL) and the combined organic fractions washed with solution of 5 % sodium bicarbonate (20 mL), 10 % hydrochloric acid solution (20.0 mL), brine (20 mL), and dried over anhydrous sodium sulfate. After evaporation of the solvent at reduced pressure, the reaction mixture was purified by chromatography on a silica gel (dichloromethane) affording the desired compound in 73 % yield. Then, 10.00 g of the mixture of acetylated cardanols was diluted with dichloromethane (20 mL) and methanol (20 mL) in a ozonolysis flask of 250 mL. The flask was adapted to the ozonator with a stream of ozone for one 1.5 h, in bath of dry ice/acetone. Next, the secondary ozonide was reduced with 5.9 g of sodium borohydride (158.7 mmol) in 60 mL of methanol. At the end of addition of sodium borohydride, the reaction remained for 6 h under stirring. Then, the mixture was acidified with concentrated hydrochloric acid to pH 3, and it was extracted with ethyl acetate (3×30 mL). The combined organic fractions were washed with brine (30 mL) and dried over sodium sulfate. After evaporation of the solvent, the product was purified by chromatography on silica gel (dichloromethane/chloroform, 5:5 and then chloroform/ethanol, 9:1) leading to the title compound **7** as colorless oil. Yield: 79 %. IR (KBr): $\tilde{\nu }$_max_=3351, 2929, 2855, 1589, 1456 cm^−1^. IV (KBr): $\tilde{\nu }$_max_=3351 (*ν*_OH_); 2929 (*ν*_as CH2_); 2855 (*ν*_s CH2_); 1589, 1456 cm^−1^ (*ν*_C=C_). ^1^H NMR (300 MHz, CDCl_3_): *δ*=1.30 (s, 8 H), 1.53–1.59 (m, 4 H), 2.54 (t, *J*=7.6 Hz, 2 H), 3.66 (t, *J*=6.6 Hz, 2 H), 6.65 (dd, *J*=8.1 Hz, *J*=2.5 Hz, 1 H), 6.67 (s, 1 H), 6,71 (d, *J*=7.6 Hz, 1 H), 7.19 ppm (d, *J*=7.8 Hz, 1 H). ^13^C NMR (75 MHz, CDCl_3_): *δ*=25.8, 29.2, 29.4, 29.5, 31.3, 32.7, 35.9, 63.2, 112.8, 115.6, 120.8, 129.5, 144.9, 156.0 ppm.

**General procedure for the synthesis of CNSL‐based hybrids (8**–**22)**: To a solution of the proper phenol derivative (**5**–**7**) (0.1 mmol and 0.3 mmol for **7**) in dry DMF (0.04 m), K_2_CO_3_ (0.1 mmol and 0.3 mmol for **7**) was added. The resulting mixture was stirred for 0.5 h at room temperature. For phenol **7**, the reaction was conducted at 0 °C to avoid the formation of side products. Subsequently, the suitable 2‐bromo‐1,4‐naphtoquinone (**23**–**27**) was added (0.1–0.3 mmol) to the reaction mixtures, which were stirred at room temperature for further 2–3 h. Then, LiCl 5 % solution was added (10 mL), whereas the reaction mixtures involving **26** and **27**, were previously acidified with a solution of 2 n HCl (pH 5), until the chemical toning changes from blue‐green to orange. Next, the obtained mixtures were extracted with ethyl acetate (10 mL×3). The organic extracts were collected, dried over Na_2_SO_4_ and the solvent was evaporated under vacuum. The crude residue was purified by chromatography on silica gel and/or purified by crystallization, when required.

**Methyl 2‐((1,4‐dioxo‐1,4‐dihydronaphthalen‐2‐yl)oxy)‐6‐pentadecylbenzoate (8)**: The title compound was obtained according to the general procedure using **5** and **23**, and purified by chromatography on silica gel with a mixture of petroleum ether/ethyl acetate (9:1), as eluent. Compound **8** was obtained as a yellow waxy solid. Yield: 61 %. ^1^H NMR (400 MHz; CDCl_3_): *δ*=0.85 (t, *J*=6.8 Hz, 2 H), 1.23–1.28 (m, 27 H), 2.66 (t, *J*=8.4 Hz, 2 H), 3.77 (s, 3 H), 5.96 (s, 1 H), 6.95 (d, *J*=8.4 Hz, 1 H), 7.18 (d, *J*=7.6 Hz, 1 H), 7.39 (t, *J*=7.6 Hz, 1 H), 7.72–7.75 (m, 2 H), 8.04–8.06 (m, 1 H), 8.15–8.16 ppm (m, 1 H). ^13^C NMR (100 MHz; CDCl_3_): *δ*=14.08, 22.65, 29.32, 29.34, 29.45, 29.50, 29.62, 29.66, 31.24, 31.89, 33.62, 52.30, 114.18, 119.04, 126.20, 126.67, 126.71, 127.76, 131.03, 131.10, 131.96, 133.48, 134.34, 143.91, 149.96, 160.16, 166.47, 179.40, 184.88 ppm. MS (ESI^+^) *m*/*z* C_33_H_42_O_5_: 541 [*M*+Na]^+^; 557 [*M*+K]^+^.

**Methyl 2‐((8‐methoxy‐1,4‐dioxo‐1,4‐dihydronaphthalen‐2‐yl)oxy)‐6 pentadecylbenzoate (9)**: The title compound was obtained according to the general procedure using **5** and **24**, and purified by chromatography on silica gel with a mixture of ethyl acetate/petroleum ether/toluene (6:3:1), as eluent. Compound **9** was obtained as a yellow waxy solid. Yield: 60 %. ^1^H NMR (400 MHz; CDCl_3_): *δ*=0.85 (t, *J*=7.2 Hz, 2 H), 1.23–1.27 (m, 27 H), 2.64 (t, *J*=8 Hz, 2 H), 3.76 (s, 3 H), 4.00 (s, 3 H), 5.87 (s, 1 H), 6.92 (d, *J*=8 Hz, 1 H), 7.15 (d, *J*=8 Hz, 1 H), 7.24–7.28 (m, 1 H), 7.37 (t, *J*=8.4 Hz, 1 H) 7.61–7.70 ppm (m, 2 H). ^13^C NMR (100 MHz; CDCl_3_): *δ*=14.08, 22.65, 29.32, 29.35, 29.44, 29.50, 29.63, 29.66, 31.24, 31.89, 33.57, 52.32, 54.50, 112.19, 117.51, 118.83, 118.87, 119.06, 126.76, 127.54, 130.97, 134.28, 135.43, 143.71, 150.13, 160.18, 160.69, 166.52, 177.77, 184.85 ppm. MS (ESI^+^) *m*/*z* C_34_H_44_O_6_: 571 [*M*+Na]^+^, 587 [*M*+K]^+^.

**Methyl 2‐((5‐methoxy‐1,4‐dioxo‐1,4‐dihydronaphthalen‐2‐yl)oxy)‐6‐pentadecylbenzoate (10)**: The title compound was obtained according to the general procedure using **5** and **25**, and purified by chromatography on silica gel with a mixture of ethyl acetate/petroleum ether/toluene (5:4:1), as eluent. Compound **10** was obtained as a yellow waxy solid. Yield: 53 %. ^1^H NMR (400 MHz; CDCl_3_): *δ*=0.84–0.87 (m, 2 H), 1.23–1.28 (m, 27 H), 2.65 (t, *J*=8 Hz, 2 H), 3.75 (s, 3 H), 3.98 (s, 3 H), 5.89 (s, 1 H), 6.94 (d, *J*=8.5 Hz, 1 H), 7.16 (d, *J*=7.6 Hz, 1 H), 7.31 (d, *J*=8.4 Hz, 1 H), 7.38 (t, *J*=8 Hz, 1 H), 7.66 (t, *J*=8 Hz, 1 H), 7.83 ppm (d, *J*=7.2 Hz, 1 H). ^13^C NMR (100 MHz; CDCl_3_): *δ*=14.10, 22.67, 29.33, 29.35, 29.47, 29.51, 29.63, 29.65, 29.67, 31.24, 31.90, 33.61, 52.26, 56.56, 116.37, 118.68, 119.06, 119.37, 119.61, 126.69, 127.55, 131.06, 133.32, 134.44, 143.79, 150.05, 158.16, 159.44, 166.55, 179.69, 184.47 ppm. MS (ESI^+^) *m*/*z* C_34_H_44_O_6_: 571 [*M*+Na]^+^, 587 [*M*+K]^+^.

**Methyl 2‐((8‐hydroxy‐1,4‐dioxo‐1,4‐dihydronaphthalen‐2‐yl)oxy)‐6‐pentadecylbenzoate (11)**: The title compound was obtained according to the general procedure using **5** and **26**, and purified by chromatography on silica gel with a mixture of petroleum ether/ethyl acetate/toluene (7:2:1), as eluent. Compound **11** was obtained as an orange–yellow waxy solid. Yield: 65 %. ^1^H NMR (400 MHz; CDCl_3_): *δ*=0.84–0.87 (m, 2 H), 1.23–1.28 (m, 27 H), 2.66 (t, *J*=8 Hz, 2 H), 3.79 (s, 3 H), 5.95 (s, 1 H), 6.95 (d, *J*=8 Hz, 1 H), 7.19 (d, *J*=8 Hz,1 H), 7.24–7.27 (m, 1 H), 7.40 (t, *J*=8 Hz, 1 H), 7.58–7.65 (m, 2 H), 11.76 ppm (s, 1 H). ^13^C NMR (100 MHz; CDCl_3_): *δ*=14.09, 22.66, 29.33, 29.45, 29.50, 29.63, 29.64, 29.66,31.24, 31.90, 33.65, 52.35, 114.26, 114.78, 118.96, 124.05, 126.60, 127.90, 131.17, 131.98, 137.14, 144.00, 149.83, 159.80, 161.93, 166.42, 183.97, 184.29 ppm. MS (ESI^+^) *m*/*z* C_33_H_42_O_6_: 557 [*M*+Na]^+^, 573 [*M*+K]^+^.

**Methyl 2‐((5‐hydroxy‐1,4‐dioxo‐1,4‐dihydronaphthalen‐2‐yl)oxy)‐6‐pentadecylbenzoate (12)**: The title compound was obtained according to the general procedure using **5** and **27**, and purified by chromatography on silica gel with a mixture of petroleum ether/ethyl acetate (8.5:1.5) as eluent. Compound **12** was obtained as an orange–yellow solid. Yield: 30 %. ^1^H NMR (400 MHz; CDCl_3_): *δ*=0.84–0.88 (m, 2 H), 1.24–1.28 (m, 28 H), 2.67 (t, *J*=8 Hz, 2 H), 3.78 (s, 3 H), 5.89 (s, 1 H), 6.95 (d, *J*=8 Hz, 1 H), 7.18–7.28 (m complex, 2 H), 7.41 (t, *J*=8 Hz, 1 H), 7.59 (t, *J*=8 Hz, 1 H), 7.70 (d, *J*=8 Hz, 1 H) 12.08 ppm (s, 1 H). ^13^C NMR (100 MHz; CDCl_3_): *δ*=14.08, 22.66, 29.34, 29.45, 29.50, 29.63, 29.66, 31.24, 31.89, 33.64, 52.36, 113.77, 114.22, 118.96, 119.59, 125.24, 126.59, 127.96, 131.01, 131.178, 135.64, 144.04, 149.84, 160.75, 161.10, 166.37, 167.537, 178.70, 190.80. MS (ESI^+^) *m*/*z* C_33_H_42_O_6_: 557 [*M*+Na]^+^, 573 [*M*+K]^+^.

**2‐(3‐Pentadecylphenoxy)naphthalene‐1,4‐dione (13)**: The title compound was obtained according to the general procedure using **6** and **23**, and purified by crystallization from EtOH/H_2_O. Compound **13** was obtained as a yellow waxy solid. Yield: 21 %. ^1^H NMR (400 MHz; CDCl_3_): *δ*=0.89 (t, *J*=8 Hz, 2 H), 1.27–1.34 (m, 27 H), 2.65 (t, *J*=4 Hz, 2 H), 5.98 (s, 1 H), 6.95–6.97 (m, 2 H), 7.14 (d, *J*=8 Hz, 1 H), 7.36 (t, *J*=4 Hz, 1 H), 7.76–7.80 (m complex, 2 H), 8.07–8.09 (m, 1 H), 8.22–8.24 ppm (m, 1 H). ^13^C NMR (100 MHz; CDCl_3_): *δ*=14.15, 14.16, 22.27, 29.24, 29.39, 29.47, 29.58, 29.68, 29.71, 29.72, 31.19, 31.95, 35.72, 113.27, 118.13, 120.88, 126.23, 126.75, 130.05, 131.11, 131.99, 133.52, 134.45, 145.96, 152.60, 160.64, 180.07, 185.11 ppm. MS (ESI^+^) *m*/*z* C_31_H_40_O_3_: 483 [*M*+Na]^+^.

**8‐Methoxy‐2‐(3‐pentadecylphenoxy)naphthalene‐1,4‐dione (14)**: The title compound was obtained according to the general procedure using **6** and **24**, and purified by chromatography on silica gel with a mixture of petroleum ether/ethyl acetate (6:4), as eluent. Compound **14** was obtained as a yellow waxy solid. Yield: 65 %. ^1^H NMR (400 MHz; CDCl_3_): *δ*=0.84–0.87 (t, *J*=8 Hz, 2 H), 1.23–1.27 (m, 27 H), 2.60 (t, *J*=7.6 Hz, 2 H), 4.02 (s, 3 H), 5.86 (s, 1 H), 6.88–6.90 (m, 2 H), 7.08 (d, *J*=8 Hz, 1 H), 7.26–7.32 (m, 2 H), 7.64–7.71 ppm (m, 2 H). ^13^C NMR (100 MHz; CDCl_3_): *δ*=14.08, 22.65, 29.19, 29.32, 29.41, 29.53, 29.63, 29.66, 31.13, 31.89, 35.68, 56.51, 111.38, 117.51, 118.13, 118.84, 118.96, 120.89, 126.48, 129.89, 134.31, 34.43, 145.77, 152.89, 160.21, 161.22, 178.33, 184.98 ppm. MS (ESI^+^) *m*/*z* C_32_H_42_O_4_: 513 [*M*+Na]^+^, 529 [*M*+K]^+^.

**5‐Methoxy‐2‐(3‐pentadecylphenoxy)naphthalene‐1,4‐dione (15)**: The title compound was obtained according to the general procedure using **6** and **25**, and purified by chromatography on silica gel with a mixture of petroleum ether/ethyl acetate (6:4), as eluent. Compound **15** was obtained as a yellow waxy solid. Yield: 30 %. ^1^H NMR (400 MHz; CDCl_3_): *δ*=0.84–0.87 (m, 2 H), 1.23–1.27 (m, 27 H), 2.60 (t, *J*=8 Hz, 2 H), 3.97 (s, 3 H), 5.87 (s, 1 H), 6.90–6.92 (m, 2 H), 7.08 (d, *J*=8 Hz, 1 H), 7.29–7.31 (m, 2 H), 7.66 (t, *J*=8 Hz, 1 H), 7.85 ppm (d, *J*=8 Hz, 1 H). ^13^C NMR (100 MHz; CDCl_3_): *δ*=14.09, 22.66, 29.19, 29.32, 29.41, 29.52, 29.63, 29.66, 31.10, 31.89, 35.68, 56.54, 115.74, 118.09, 118.70, 119.38, 119.64, 120.84, 126.47, 129.92, 133.40, 134.39, 145.80, 152.70, 158.57, 159.40, 180.27, 180.51 ppm. MS (ESI^+^) *m*/*z* C_32_H_42_O_4_: 513 [*M*+Na]^+^, 529 [*M*+K]^+^.

**8‐Hydroxy‐2‐(3‐pentadecylphenoxy)naphthalene‐1,4‐dione (16)**: The title compound was obtained according to the general procedure using **6** and **26**, and purified by crystallization from EtOH/H_2_O. Compound **16** was obtained as an orange waxy solid. Yield: 45 %. ^1^H NMR (400 MHz; CDCl_3_): *δ*=0.86–0.89 (m, 2 H), 1.25–1.31 (m, 27 H), 2.63 (t, *J*=7.6 Hz, 2 H), 5.95 (s, 1 H), 6.92–6.94 (m, 2 H), 7.12 (d, *J*=7.6 Hz, 1 H), 7.28 (m, H), 7.35 (t, *J*=8 Hz, 1 H), 7.58–7.66 (m, 2 H), 11.79 ppm (s, 1 H). ^13^C NMR (100 MHz; CDCl_3_): *δ*=14.04, 22.63, 29.16, 29.30, 29.38, 29.50, 29.60, 29.64, 31.07, 31.87, 35.66, 113.86, 114.32, 117.94, 118.90, 120.69, 123.98, 126.79, 130.06, 132.01, 137.15, 146.02, 152.51, 160.23, 161.97,184.06, 184.78 ppm. MS (ESI^+^) *m*/*z* C_31_H_40_O_4_: 499 [*M*+Na]^+^, 515 [*M*+K]^+^.

**5‐Hydroxy‐2‐(3‐pentadecylphenoxy)naphthalene‐1,4‐dione (17)**: The title compound was obtained according to the general procedure using **6** and **27**, and purified by chromatography on silica gel with a mixture of petroleum ether/toluene/ethyl acetate (8.5:1:0.5), as eluent. Compound **17** was obtained as an orange waxy solid. Yield: 3 %. ^1^H NMR (400 MHz; CDCl_3_): *δ*=0.85 (t, *J*=7.2 Hz, 2 H), 1.23–128 (m, 27 H), 2.64 (t, *J*=7.6 Hz, 2 H), 5.88 (s, 1 H), 6.93–6.95 (m, 2 H), 7.14 (d, *J*=8 Hz, 1 H), 7.30 (d, *J*=8.4 Hz, 1 H), 7.36 (t, *J*=8 Hz, 1 H), 7.62 (t, *J*=8 Hz, 1 H), 7.75 (d, *J*=8 Hz, 1 H), 12.13 ppm (s, 1 H). ^13^C NMR (100 MHz; CDCl_3_): *δ*=14.05, 22.64, 29.16, 29.30, 29.39, 29.50, 29.61, 29.64, 31.07, 31.87, 35.65, 112.85, 114.19, 117.95, 119.59, 120.71, 125.24, 126.83, 130.05, 131.10, 135.56, 146.01, 152.53, 161.09, 161.19, 179.23, 190.90 ppm. MS (ESI^+^) *m*/*z* C_31_H_40_O_4_: 499 [*M*+Na]^+^.

**2‐(3‐(8‐Hydroxyoctyl)phenoxy)naphthalene‐1,4‐dione (18)**: The title compound was obtained according to the general procedure using **7** and **23**, and purified by chromatography on silica gel with a mixture of *n*‐hexane/ethyl acetate (5:5), and then by crystallization from *n*‐hexane/EtOH. Compound **18** was obtained as an orange‐yellow waxy solid. Yield: 25 %. ^1^H NMR (400 MHz; CDCl_3_): *δ*=1.32–1.52 (m, 10 H), 1.53–1.61 (m, 6 H), 2.63 (t, *J*=7.6 Hz, 2 H), 3.63 (t, *J*=6.4, 2 H), 5.96 (s, 1 H), 6.93–6.95 (m, 2 H), 7.11 (d, *J*=7.6 Hz, 1 H), δ7.35 (t, *J*=8 Hz, 1 H), 7.74–7.78 (m, 2 H), 8.05–8.07 (m, 1 H), 8.19–8.21 ppm (m, 1 H). ^13^C NMR (100 MHz; CDCl_3_): *δ*=14.09, 22.61, 25.64, 28.97, 29.24, 29.32, 31.01, 31.55, 32.72, 35.62, 62.96, 113.21, 118.10, 120.81, 126.18, 126.70, 126.75, 130.04, 131.07, 131.94, 133.48, 134.39, 145.78, 152.59, 160.60, 179.16, 185.07 ppm. MS (ESI^+^) *m*/*z* C_24_H_26_O_4_: 401 [*M*+Na]^+^.

**2‐(3‐(8‐Hydroxyoctyl)phenoxy)‐8‐methoxynaphthalene‐1,4‐dione (19)**: The title compound was obtained according to the general procedure using **7** and **24**, and purified by chromatography on silica gel with a mixture of ethyl acetate/petroleum ether/toluene/EtOH (5.5:3:1:0.5). Compound **19** was obtained as an orange‐yellow waxy solid. Yield: 20 %. ^1^H NMR (400 MHz; CDCl_3_): *δ*=1.32 (s, 10 H), 1.54–1.62 (m complex, 6 H), 2.62 (t, *J*=7.6 Hz, 2 H), 3.63 (t, *J*=6.4 Hz, 2 H), 4.04 (s, 3 H), 5.87 (s, 1 H), 6.91–6.92 (m, 2 H), 7.19 (d, *J*=7.6 Hz, 1 H), 7.29–7.33 (m, 2 H), 7.68–7.71 ppm (m, 2 H). ^13^C NMR (100 MHz; CDCl_3_): *δ*=25.61, 28.94, 29.20, 29.27, 30.94, 32.74, 35.59, 56.50,62.96, 111.43, 117.59, 118.12, 118.85, 120.84, 126.44, 129.89, 134.36, 135.35, 145.64, 152.99, 160.25, 161.22, 178,23, 184.94 ppm. MS (ESI^+^) *m*/*z* C_25_H_28_O_5_: 431 [*M*+Na]^+^, 447 [*M*+K]^+^.

**2‐(3‐(8‐Hydroxyoctyl)phenoxy)‐5‐methoxynaphthalene‐1,4‐dione (20)**: The title compound was obtained according to the general procedure using **7** and **25**, and purified by chromatography on silica gel with a mixture of ethyl acetate/*n*‐hexane/toluene (7:2:1). Compound **20** was obtained as an orange–yellow waxy solid. Yield: 34 %. Compound **20** was also synthesized from **7** (0.3 mmol), **25** (0.3 mmol), and K_2_CO_3_ (0.3 mmol) in dry DMSO (0.04 m), and purified according to the above‐reported procedure. Yield: 31 %. ^1^H NMR (400 MHz; CDCl_3_): *δ*=1.23–1.29 (m, 8 H), 1.51–1.63 (m, 6 H), 2.60 (t, *J*=7.6, 2 H), 3.61 (t, *J*=6.8 Hz, 2 H), 3.97 (s, 3 H), 5.86 (s, 1 H), 6.90–6.91 (m, 2 H), 7.07 (d, *J*=7.6 Hz, 1 H), 7.29–7.33 (m, 2 H), 7.66 (t, *J*=8 Hz, 1 H), 7.84–7.86 ppm (m, 1 H). ^13^C NMR (100 MHz; CDCl_3_): *δ*=25.62, 28.94, 29.24, 29.32, 30.97, 32.75, 35.59, 56.53,62.96, 115.44, 118.14, 118.72, 119.33, 119.65, 120.85, 126.52, 129.97, 133.38, 134.43, 145.66, 152.69, 158.61, 159.41, 180.27, 184.67 ppm. MS (ESI^+^) *m*/*z* C_25_H_28_O_5_: 431 [*M*+Na]^+^, 447 [*M*+K]^+^.

**8‐Hydroxy‐2‐(3‐(8‐hydroxyoctyl)phenoxy)naphthalene‐1,4‐dione (21)**: The title compound was obtained according to the general procedure using **7** and **26**, and purified by chromatography on silica gel with a mixture of ethyl acetate/*n*‐hexane/toluene (5:4:1). Compound **21** was obtained as an orange–yellow waxy solid. Yield: 13 %. ^1^H NMR (400 MHz; CDCl_3_): *δ*=1.26–1.32 (m, 11 H), 1.54–1.64 (m complex, 8 H), 2.64 (t, *J*=7.6 Hz, 2 H), 3.63 (t, *J*=6.6 Hz, 2 H), 5.95 (s, 1 H), 6.93–6.95 (m, 2 H), 7.13 (d, *J*=7.6 Hz, 1 H), 7.2–7.29 (m, 1 H), 7.36 (t, *J*=7.6 Hz, 1 H), 7.59–7.67 (m, 2 H), 11.80 ppm (s, 1 H). ^13^C NMR (100 MHz; CDCl_3_): *δ*=25.64, 28.98, 29.24, 29.32, 31.01, 32.74, 35.62, 63.00, 113.82, 114.32, 118.02, 118.94, 120.72, 124.02, 126.83, 130.11, 131.99, 137.21, 145.91, 152.49, 160.27, 161.98, 184.12, 184.79 ppm. MS (ESI^+^) *m*/*z* C_24_H_26_O_5_: 417 [*M*+Na]^+^, 433 [*M*+K]^+^.

**5‐Hydroxy‐2‐(3‐(8‐hydroxyoctyl)phenoxy)naphthalene‐1,4‐dione (22)**: The title compound was obtained according to the general procedure using **7** and **27**, and purified by chromatography on silica gel with a mixture of ethyl acetate/*n*‐hexane/toluene (5:4:1). Compound **22** was obtained as an orange–yellow waxy solid. Yield: 28 %. ^1^H NMR (400 MHz; CDCl_3_): *δ*=1.30–1.40 (m, 12 H), 1.52–1.59 (m complex, 5 H), 2.61 (t, *J*=7.6 Hz, 2 H), 3.61 (t, *J*=6.6 Hz, 2 H), 5.87 (s, 1 H), 6.91–6.92 (m, 2 H), 7.11 (d, *J*=7.6 Hz, 1 H), 7.24–7.33 (m, 2 H), 7.58 (t, *J*=8 Hz, 1 H), 7.71 (d, *J*=7.2 Hz, 1 H), 12.09 ppm (s, 1 H). ^13^C NMR (100 MHz; CDCl_3_): *δ*=25.65, 29.00, 29.25, 31.02, 32.73, 35.62, 63.01, 112.82, 114.17, 118.01, 119.65, 120.72, 125.30, 126.87, 130.10, 131.07, 135.62, 145.91, 152.49, 161.07, 161.19, 179.26, 190.53 ppm. MS (ESI^+^) *m*/*z* C_24_H_26_O_5_: 417 [*M*+Na]^+^, 433 [*M*+K]^+^.

### Prediction of physicochemical properties and pan‐assay interference compounds (PAINS) analysis

The online server FAF*Drugs*4^43^ (http://fafdrugs3.mti.univ‐paris‐diderot.fr) was used to predict physicochemical properties of **8**–**22** (Supporting Information Table S1). These include: number of rotatable bonds, hydrogen‐bond acceptors (HBAs), hydrogen‐bond donors (HBDs), MW, log*P*, log*D* (at pH 7), topological polar surface area (tPSA), flexibility, and aqueous solubility (log*S*_w_). FAF*Drugs*4^43^ was also used to screen **8**–**22** for known classes of pan‐assay interference compounds (PAINS). As expected, **8**–**22** were flagged as potential PAINS, due to the presence of the quinone sub‐structure and the long alkyl chain. However, despite the potential for these compounds to interfere with non‐cellular assays,^44^ we found no activity against isolated *Tb*GAPDH protein. Furthermore, the fact that the trypanocidal activity is limited to quinones with C_8_ alkyl chain (**18**–**22**) points to a high degree of specificity and interaction with well‐defined target(s) rather than a nonspecific interaction. In addition, **20** and **22** showed severalfold lower trypanocidal activity when evaluated against *T. congolense* (IL3000 WT). These observations led us to believe that the current subset does not behave as PAINS.

### Biological evaluation

**Organisms and culture media**: Only bloodstream trypomastigotes of *T. b. brucei* were used throughout this study. The drug‐sensitive wild‐type strain *Trypanosoma brucei brucei* Lister 427 (427WT)25a was used alongside three multidrug‐resistant strains: B48, ISMR1, and aqp2/aqp3‐KO. B48 was created from 427WT after deletion of the *TbAT1* gene, encoding for the P2 drug transporter,25b and adaptation to increasing concentrations of PMD.^45^ The aqp2/aqp3‐KO strain was generated from wild‐type *T. b. brucei* 2T1 cells after knockout of the locus encoding for the aquaglyceroporin 2 and 3 channels, resulting in melarsoprol–pentamidine cross‐resistance.^21^, ^46^ ISMR1 is an ISM‐resistant clone obtained from *T. b. brucei* 427WT that lost their kinetoplast DNA and express an F_o_F_1_‐ATP synthase mutation.^32^ All the *T. b. brucei* strains were cultured as described^47^ in standard HMI‐9 medium, supplemented by 10 % of heat‐inactivated fetal bovine serum (FBS), 14 μL L^−1^ of β‐mercaptoethanol, and 3.0 g L^−1^ of sodium hydrogen carbonate (pH 7.4). Parasites were cultured in vented flasks at 37 °C and 5 % CO_2_ atmosphere and they were passaged every 3 days. Bloodstream forms of *T. congolense* savannah‐type strain IL3000 were cultured in basal MEM medium, supplemented by 10 % fresh goat serum, 14 μL of β‐mercaptoethanol, 800 μL of 200 mm glutamine solution, and 10 mL of penicillin/streptomycin solution per liter of medium (pH 7.3).^48^ *T. congolense* were cultured in six‐well plates at 34 °C and 5 % CO_2_.

**In vitro drug susceptibility assay**: The drug susceptibilities of bloodstream‐form trypanosomes of *T. b. brucei* 427WT, B48, aqp2/aqp3‐KO and ISMR1 strains were determined by using the resazurin (Alamar blue) viability indicator dye following a previously described protocol,^49^ slightly adapted for *T. congolense*. In brief, the assays were performed in 96‐well plates with 2×10^5^ cells per well for *T. b. brucei* or 5×10^5^ cells per well for *T. congolense*, in their respective culture media. First, 200 μL of test compounds’ solutions (400 μm, in *T. brucei* or *T. congolense* medium as appropriate) was added to the first well of each 12‐well row, from which doubling dilutions were conducted over one row per test compound. Trypanosomes (100 μL in each well) were added and the plates were incubated for 48 h at 37 °C and 5 % CO_2_, followed by addition of 20 μL Alamar blue solution (125 mg mL^−1^ of resazurin sodium salt (Sigma–Aldrich) in phosphate‐buffered saline (PBS)) followed by 24 h of incubation at 37 °C and 5 % CO_2_. PMD (Sigma–Aldrich) (for 427WT, B48, aqp2/aqp3‐KO), DA (Sigma–Aldrich; for *T. congolense*) and ISM (gift from Merial France) (for ISMR1) were used as trypanocidal positive controls. Fluorescence was detected using a FLUOstar Optima (BMG Labtech, Durham, NC, USA) at wavelength of 540 nm (excitation), 590 nm (emission). EC_50_ values were calculated by nonlinear regression using an equation for a sigmoidal dose–response curve with variable slope using Prism 5.0 (GraphPad Software Inc., San Diego, CA, USA).

**Growth curve**: *T. b. brucei* 427WT cells were grown to mid‐log‐phase in standard HMI‐9/FBS medium distributed in in six‐well plates at 1×10^5^ cells mL^−1^ and incubated with two different concentrations of test compounds (EC_50,_ 2×EC_50_) for 48 h at 37 °C and 5 % CO_2_. Untreated parasites, used as control, were grown in parallel. The cells were counted by using hemocytometer cell counter (cell count/mL×10^4^) at 0, 2, 4, 8, 12, 24, 28, 32, 36, 48 h of incubation. Each experiment was performed as two independent replicates, and each sample was counted at least twice.

**Cytotoxicity assay on human foreskin fibroblast (HFF)**: Toxicity of test compounds to mammalian cells was carried out using the resazurin assay previously described^31^ with slight modifications as follows. HFF cells were grown in a culture medium containing 500 mL of Dulbecco's modified Eagle's medium (DMEM; Sigma), 50 mL newborn calf serum (NBCS; Gibco), 5 mL penicillin/streptomycin (Gibco), and 5 mL of l‐Glutamax (200 nm, Gibco), at 37 °C and 5 % CO_2_ in vented flasks and passaged at 80–85 % of confluence. For the cytotoxicity assay, cells were suspended at 3×10^5^ cells mL^−1^ and 100 μL aliquots were added to each well of a 96‐well plate. The plate was incubated at 37 °C and 5 % CO_2_ for 24 h to allow cell adhesion. Serial test compounds dilution was performed in a different 96‐well plate and 100 μL of each dilution was transferred to each well containing cells, resulting in exposure of cells to 200–0.2 μm of test compound. Phenylarsine oxide (Sigma–Aldrich) was used as positive control and drug‐free incubation as negative control. The plates were incubated at 37 °C and 5 % CO_2_ for an additional 30 h, at which point 10 μL of resazurin solution (125 mg mL^−1^ in PBS) was added, followed by a final incubation for 24 h. The plates were read and the data analyzed as described above for the trypanosome assay.

**ATP assay on** ***T. b. brucei*** **and HFF cells**: Changes in cellular ATP levels due to the exposure of trypanosomes to the test compounds (0.5×EC_50_) were monitored using the Molecular Probes ATP Determination Kit (A22066, Invitrogen Detection Technologies), based on the luciferin–luciferase bioluminescent enzymatic reaction. Bloodstream trypomastigote cultures of *T. brucei* s427WT were incubated with and without test compound, and, at each predetermined incubation time, 10^7^ cells of each sample were transferred into a microfuge tube and centrifuged at 2000×*g* for 10 min at 4 °C. The pellet was washed twice with 1 mL of 50 mm Tris⋅HCl (pH 7.4) containing 0.1 mm DTT, and then resuspended in 200 μL of the same buffer. The cells were lysed by sonication on ice (twice for 10 s separated by 30 s), using a Soniprep 150 (MSE) at 8 μm amplitude. The samples were centrifuged at 10 000×*g* for 10 min at 4 °C and the supernatant was collected, instantly frozen in liquid nitrogen, and stored at −80 °C. Oligomycin (2.0 μg mL^−1^) was used as positive control. ATP levels were quantified using the contents of the kit following the manufacturer's instructions; 90 μL of standard reaction solution was added to each well of a 96‐well plate and the background luminescence was recorded in a FLUOstar OPTIMA fluorimeter; 10 μL of each sample was then added to each well. The plate was incubated at 28 °C for 15 min and the luminescence was measured, including a standard curve with an ATP concentration ranging from 1 nm to 1 μm to allow the calculation of the ATP concentrations in each sample. The ATP content was measured at 0, 0.5, 1, 2, 4, 8 and 12 h of incubation with test sample.

The same procedure was used to determine the ATP content of HFF cells, using 200 μm of the test compounds for incubation times of 0, 4, and 8 h, 1×10^6^ HFF cells per sample and a centrifugation speed for pelleting the cells of 800×*g* for 5 min. Oligomycin (2 μg mL^−1^) was used as a positive control as described.^50^

**Mitochondrial membrane potential assay on** ***T. b. brucei***: Changes in mitochondrial membrane potential (*Ψ*_m_) after incubation of trypanosomes with the test compounds were determined using fluorescence‐activated cell sorting (FACS) with the indicator dye tetramethylrhodamine ethyl ester (TMRE), as described^34^ with minor modifications. Cell suspensions of *T. b. brucei* 427WT trypomastigotes were incubated at 0.5×EC_50_ with test compound and 1×10^7^ cells were transferred at each time point (0, 0.5, 1, 2, 4, 8 and 12 h) into a microfuge tube and centrifuged at 2000×*g* for 10 min at room temperature. The pellet was washed once in 1 mL of PBS (pH 7.4) and resuspended in 1 mL of PBS containing 200 nm TMRE; the cells then were incubated at 37 °C for 30 min and subsequently placed on ice for another 30 min before analysis on a Becton Dickinson FACSCalibur using a FL2‐height detector, and CellQuest and FlowJo software. Valinomycin (Sigma–Aldrich; 100 nm) and troglitazone (Sigma–Aldrich; 10 μm) were used as controls for mitochondrial membrane depolarization and positive hyperpolarization, respectively.^51^

**Transmission electron microscopy assay on** ***T. b. brucei*** **and HFF cells**: TEM of bloodstream trypomastigotes (427WT) and of HFF was performed essentially as previously described.^52^ Briefly, cell cultures were adjusted to 2.5×10^6^ cells mL^−1^ and incubated in the presence or absence of test compounds at EC_50_. Cells were fixed overnight at 4 °C in 2.5 % glutaraldehyde and 4 % paraformaldehyde in 0.1 m phosphate buffer (pH 7.4). Samples were washed with 0.1 m phosphate buffer (pH 7.4), post‐fixed in 1 % osmium tetroxide for 1 h on ice, and washed with the phosphate buffer. Cells were next incubated in 0.5 % uranyl acetate solution for 30 min, washed with distilled water and dehydrated in increasing concentrations of acetone (30, 50, 70, 90 and 100 %). Cells were embedded in epoxy resin; thin sections of 50–60 nm were observed in a Tecnai T20 (FEI) at 200 kV.

**Reactive oxygen species (ROS) assay on** ***T. b. brucei***: The production of ROS when *T. b. brucei* 427WT trypomastigotes incubated with different concentration (2.5×EC_50_; 1.25×EC_50_; 0.3×EC_50_) of test compound was assessed using the indicator dye 2,7‐dichlorodihydrofluorescein diacetate (DCFH‐DH; Sigma–Aldrich). This assay was performed in a 96‐well black‐bottomed well plate; 200 μL of each test compound solution, at 5×EC_50_ in assay buffer pH 7.3, was added to the well in the first column of the plate and doubling dilutions were carried out across the row, after which 3×10^6^ cell in 100 μL of assay buffer were added to each well, immediately followed by 2 μL of 1 mm DCFH‐DH, under minimal light conditions. The plates were incubated in a FLUOstar OPTIMA fluorimeter at 37 °C 5 % CO_2_ and the fluorescence was monitored at 485 nm for the excitation, 520 nm for the emission for 3 h, taking readings of each well every 2 min. Three wells were included in the plate as controls: 1) 3×10^6^ cells per well in assay buffer without test compound; 2) 3×10^6^ cells per well in assay buffer with 2 μL of 10 mm H_2_O_2_; 3) assay buffer without cells and test compound.

**Metabolomics assay in** ***T. b. brucei***: The experiment was performed to detect changes in the intracellular metabolic pathway in *T. b. brucei* 427WT BSF after 2 h incubation with the most active test compound, at a concentration of 0.5×EC_50_, and compared with a DMSO drug free control. Parasites were grown to 1.5×10^6^ cells mL^−1^ in order to have a mid‐log‐phase culture at the end of the incubation time. At the end of the incubation, the cell density was adjusted and 1×10^8^ cells were transferred to a 50 mL centrifuge tube. The samples were quenched by rapidly cells cooling to 4 °C in a dry ice/ethanol bath and centrifuged at 1250×*g* for 10 min at the same temperature. 10 μL of supernatant (spent medium) was collected, 200 μL of CMW (chloroform/methanol/water 1:4:1) was added and the mixture stored at −80 °C until LC–MS analysis. The rest of the supernatant was discarded; the cell pellet was transferred to a microfuge tube and centrifuged at 4500 rpm for 5 min at 4 °C. The pellet was washed once in 1 mL PBS at 4 °C and resuspended in 200 μL of CMW extraction solvent at 4 °C. The samples were shaken at 4 °C for 1 h to break up the pellet and allow the complete extraction of intracellular metabolites, followed by centrifugation at 13 000 rpm for 10 min at 4 °C. 180 μL of supernatant was collected in an LC–MS vial. For each biological replicate, 15 μL from each sample were all combined in the same MS vial; this pooled sample served as a quality control. All samples were gassed with Ar before sealing and stored at −80 °C. All the samples were analyzed with LC–MS. The experiment was conducted in three independent biological replicates, each analyzed in duplicate (two technical replicates).

**LC–MS analysis and data extraction**: Samples were randomly placed in the autosampler tray and the LC–MS experiment was performed on an Accela 600 HPLC system combined with an Exactive (Orbitrap) mass spectrometer from Thermo Fisher Scientific (Hemel Hempstead, UK). In separate runs, 10 μL of sample was injected onto a HiChrom Ltd. (Reading, UK) ZIC‐pHILIC column (150×4.6 mm, 5 μm particle size). The LC–MS system was run in binary gradient mode; a flow rate of 0.3 mL min^−1^ was used and samples were kept in a vial tray set at 3 °C. The gradient conditions were as follows: (A) 20 mm ammonium carbonate pH 9.2, (B) acetonitrile; 0 min 80 % B; 30 min 20 % B; 36 min 20 % B, 37 min 80 % B; 46 min 80 % B. The ESI interface was operated in positive and negative ion switching mode, with +4.0 kV of spray voltage for positive mode and −3.5 kV for negative mode. The temperature of the ion‐transfer capillary was 270 °C and sheath and auxiliary gas were set at 57 and 17 arbitrary units, respectively. The full scan range of both positive and negative modes was set at 75 to 1200 *m*/*z* with AGC target and resolution as Balanced and High (1E6 and 50 000), respectively. Prior to analysis, mass calibration was performed for both ESI modes using the standard Thermo Calmix solution. The mass spectrometry data was extracted by using *m*/*z* Mine 2.20^53^ and the masses were searched against an in‐house database.

## Conflict of interest

*The authors declare no conflict of interest*.

## Supporting information

As a service to our authors and readers, this journal provides supporting information supplied by the authors. Such materials are peer reviewed and may be re‐organized for online delivery, but are not copy‐edited or typeset. Technical support issues arising from supporting information (other than missing files) should be addressed to the authors.

Supplementary
