# Supplementary material for: Discovery of Sustainable Drugs for Neglected Tropical Diseases: Cashew Nut Shell Liquid (CNSL)‐Based Hybrids Target Mitochondrial Function and ATP Production in Trypanosoma brucei
Source: ChemMedChem. 2019 Feb 5;14(6):621–35. doi: 10.1002/cmdc.201800790 (PMC6686156; doi:10.1002/cmdc.201800790)
Supplement: Supplementary file 1 — Supplementary [file CMDC-14-621-s001.pdf]

CHEMMEDCHEM
Chemistry & Drug Discovery

Supporting Information

Discovery of Sustainable Drugs for Neglected Tropical Diseases: Cashew Nut Shell Liquid (CNSL)-Based Hybrids Target Mitochondrial Function and ATP Production in *Trypanosoma brucei*

Michela Cerone,[a, b] Elisa Uliassi,[a] Federica Prati,[a] Godwin U. Ebiloma,[b, c] Leandro Lemgruber,[b, d] Christian Bergamini,[a] David G. Watson,[e] Thais de A. M. Ferreira,[f] Gabriella Simões Heyn Roth Cardoso,[f] Luiz A. Soares Romeiro,[f] Harry P. de Koning,[b] and Maria Laura Bolognesi*[a]

cmdc_201800790_sm_miscellaneous_information.pdf

Table of contents

Fig. S1 S2

Fig. S2 S3

Fig. S3 S4

Table S1 S5

Compound purity and Table S2 S6

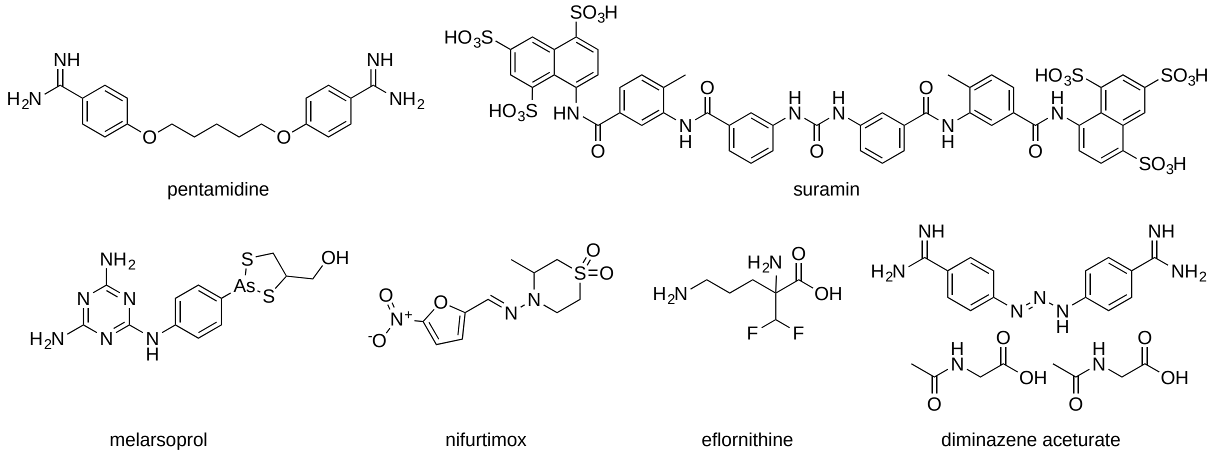

Fig. S1. Available drugs for the treatment of human African trypanosomiasis (HAT) and animal African trypanosomiasis (AAT).

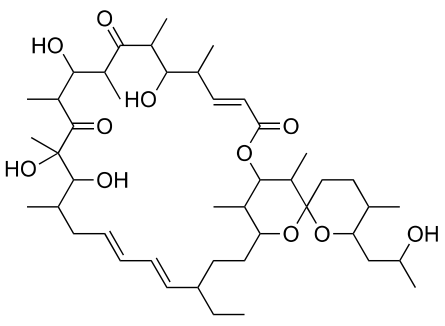

oligomycin

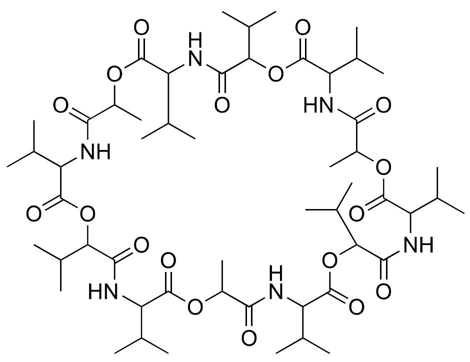

valinomycin

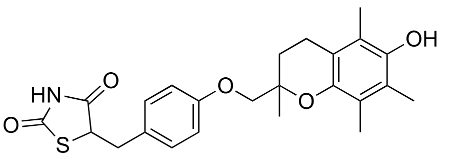

troglitazone

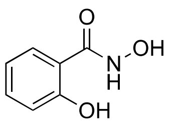

salicylhydroxamic acid

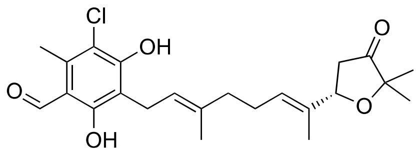

ascofuranone

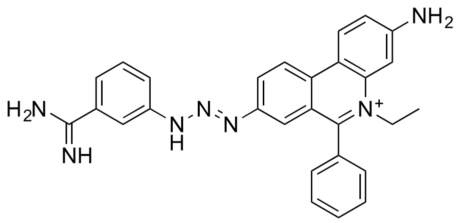

isometamidium

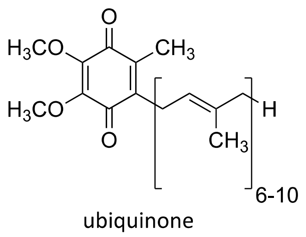

ubiquinone

Fig. S2. Positive and negative controls used in the experiments aimed at determining the mitochondrial effects of the hybrids.

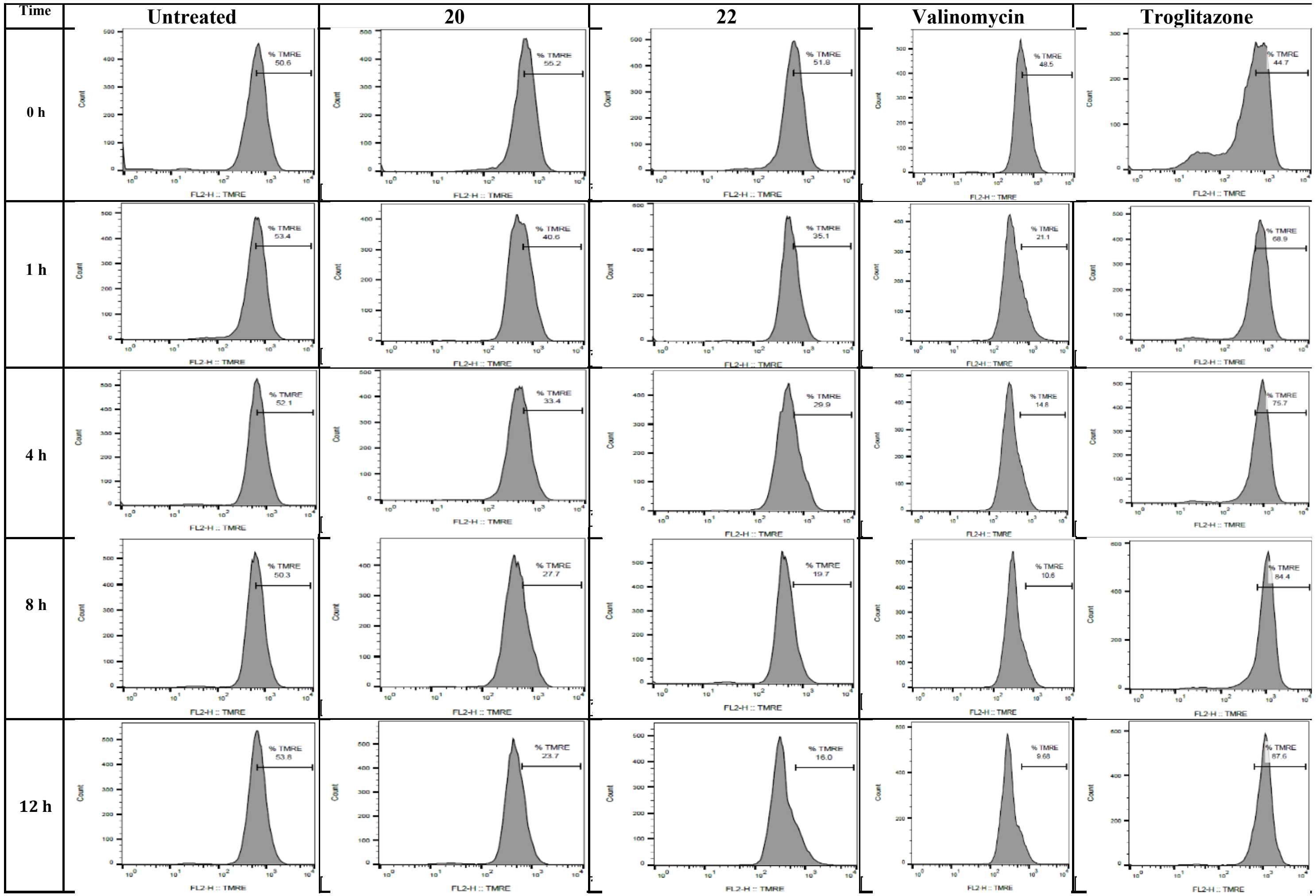

Fig. S3. Histograms of one sample determination of TMRE (200 nM) fluorescence in *T. b. brucei* s427WT BSF treated with **20** and **22** (½ EC_{50}). Values are given as the percentage of cells with fluorescence above 500 arbitrary units (A. U.), which was set at approximately 50% for the untreated control at t = 0 h. A shift to higher fluorescence represents an increasing of MMP because of a stronger accumulation of TMRE in the mitochondrion; lower values represent a decreasing of MMP.

Table S1. Physico-chemical properties of **8-22** predicted with FAF-Drugs4 software (http://fafdrugs3.mti.univ-paris-diderot.fr).

	MW	logP	logD	logSw	tPSA	RotatableB	Flexibility	HBD	HBA
**8**	518.68	10.88	9.52	-8.83	69.67	18	0.47	0	5
**9**	548.71	10.85	9.36	-8.93	78.90	19	0.49	0	6
**10**	548.71	10.85	9.36	-8.93	78.90	19	0.49	0	6
**11**	534.68	11.07	9.86	-9.05	89.90	18	0.47	1	6
**12**	534.68	11.07	9.86	-9.05	89.90	18	0.47	1	6
**13**	460.65	10.75	9.52	-8.53	43.37	16	0.46	0	3
**14**	490.67	11.0	9.36	-8.8	52.60	17	0.47	0	4
**15**	490.67	11.0	9.36	-8.8	52.60	17	0.47	0	4
**16**	476.65	11.22	9.86	-8.93	52.60	16	0.46	1	4
**17**	476.65	11.22	9.86	-8.93	63.60	16	0.46	1	4
**18**	378.46	5.64	4.97	-5.24	63.60	10	0.34	1	4
**19**	408.49	5.62	4.81	-5.34	72.83	11	0.37	1	5
**20**	408.49	5.62	4.81	-5.34	72.83	11	0.37	1	5
**21**	394.46	5.84	5.31	-5.47	83.83	10	0.34	2	5
**22**	394.46	5.84	5.31	-5.47	83.83	10	0.34	2	5

Compound purity

The purity of **8-22** was determined using Kinetex® 5µm EVO C18 100 Å, LC Column 150 x 4.6 mm and HPLC Jasco Corporation (Tokyo, Japan) instrument, model PU-1585 UV equipped with a 20 µL loop valve. HPLC parameters were the following: MeOH (eluent A) and H_2O with 0.05% trifluoroacetic acid (eluent B); flow rate 1.0 mL/min; elution type isocratic with 50% of eluent A and 50% of eluent B; detection UV-Vis Abs at 254 nm.

The samples were dissolved in MeOH (10 µg/mL).

Table S2. Compound purity by HPLC.

Entries	Purity (%)	t_R	Entries	Purity (%)	t_R	Entries	Purity (%)	t_R
**8**	97	3.0	**13**	95	3.0	**18**	100	2.9
**9**	96	3.3	**14**	99	3.5	**19**	100	3.1
**10**	95	3.5	**15**	95	3.3	**20**	97	3.0
**11**	100	4.2	**16**	96	4.1	**21**	100	3.9
**12**	95	4.2	**17**	95	4.2	**22**	100	4.1
